# Capsular polysaccharide-mediated protein loading onto extracellular membrane vesicles of *Shewanella vesiculosa* HM13

**DOI:** 10.3389/fmicb.2026.1934777

**Published:** 2026-09-09

**Authors:** Kouhei Kamasaka, Jun Kawamoto, Taiku Tsudzuki, Yuanzheng Yang, Yuying Liu, Tomoya Imai, Angela Casillo, Takuya Ogawa, Maria Michela Corsaro, Tatsuo Kurihara

**Affiliations:** 1 Institute for Chemical Research, Kyoto University, Gokasho, Uji, Kyoto, Japan; 2 Research Institute for Sustainable Humanosphere, Kyoto University, Gokasho, Uji, Kyoto, Japan; 3 Department of Chemical Sciences, University of Naples “Federico II”, Complesso Universitario Monte S. Angelo, Naples, Italy

**Keywords:** extracellular vesicles, Gram-negative bacteria, outer membrane, polysaccharide, protein secretion

## Abstract

Bacterial extracellular membrane vesicles (EMVs) play crucial physiological roles, including intercellular communication and nutrient acquisition, which depend on the functions of their cargo molecules. However, the molecular mechanisms governing selective cargo loading onto EMVs remain poorly understood. Here, we investigated the role of capsular polysaccharide (CPS) in the association of P49, the major cargo protein of EMVs from *Shewanella vesiculosa* HM13, with EMVs. Deleting genes neighboring the P49 gene led to a loss of EMV-associated CPS and a defect in P49 loading. *In vitro* binding assays using P49-free EMVs and purified P49 revealed that exogenously added P49 specifically binds to CPS-containing EMVs, but not to CPS-deficient EMVs. Furthermore, gel-filtration analysis demonstrated that purified P49 directly binds to CPS extracted from cells. Transmission electron microscopy revealed that P49 constitutes peripheral nanoparticles on EMV surfaces, which were absent in CPS-deficient mutants. These findings identify CPS as a key factor required for P49 association with EMVs and support a model in which CPS functions as a scaffold for cargo protein loading. This P49–CPS system provides a basis for future mechanistic studies of P49 loading onto EMVs and may contribute to the development of post-vesiculation protein display strategies using bacterial EMVs.

## 1 Introduction

Extracellular membrane vesicles (EMVs) released from bacteria are nano-sized spherical bilayer structures typically ranging in size from 20 to 250 nm. EMVs play diverse roles in bacterial physiology, including intra- and inter-species communication, horizontal gene transfer, pathogenesis, biofilm formation, and self-defense (Caruana and Walper, 2020; Toyofuku et al., 2015). These functions of EMVs depend on their cargo molecules. For instance, EMVs of pathogenic bacteria such as *Pseudomonas aeruginosa*, *Vibrio cholerae*, and enterotoxigenic *Escherichia coli* selectively deliver multiple virulence factors for survival and proliferation within the host, which may result in severe disease (Kadurugamuwa and Beveridge, 1995; Chatterjee and Chaudhuri, 2011; Kunsmann et al., 2015). Other examples include EMVs carrying hydrophobic signaling molecules, which play important roles in quorum sensing, and those harboring β-lactamases, which contribute to antibiotic resistance (Caruana and Walper, 2020). Loading onto EMVs provides cargo proteins with various advantages, including protection from proteolytic degradation, long-distance delivery, host-cell specific targeting, and modulation of the immune response (Bonnington and Kuehn, 2014). In addition to their physiological importance, vesiculation-mediated protein secretion has potential for various applications, such as developing drug delivery systems, nanobiocatalysts, vaccines, and heterologous protein production systems (Toyofuku et al., 2015). Thus, cargo loading onto EMVs is important for their physiological functions and potential applications.

**Figure 1:**
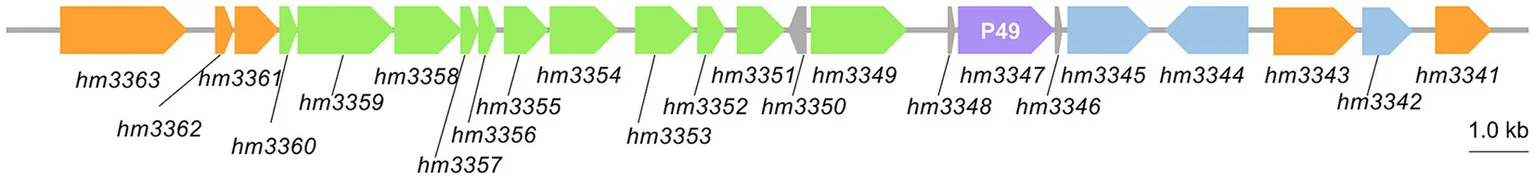
Genetic organization of the P49 gene-containing gene cluster. Genetic map of the gene cluster containing the P49 gene and its neighboring genes. Predicted functions and localization of the gene products are listed in Supplementary Table 1. The P49-coding gene is indicated by the purple arrow, while genes having homology to genes involved in polysaccharide synthesis and the T2SS-coding genes are indicated by orange and green arrows, respectively. Genes coding for homologs of glycerophosphodiester phosphodiesterase, phosphoethanolamine transferase, and nitroreductase are shown by blue arrows.

*Shewanella vesiculosa* HM13, a fish intestinal cold-adapted Gram-negative bacterium, produces abundant EMVs carrying a single major cargo protein named P49 (Chen et al., 2019). Although the physiological function of P49 is yet to be elucidated and is unpredictable due to a lack of sequence similarity to known proteins, the high quantity of P49 in EMVs raises the expectation that it can be used as a carrier protein to deliver a foreign protein to EMVs as a major cargo using a recombinant protein production system (Chen et al., 2019; Kawamoto and Kurihara, 2022). Our previous study demonstrated that a gene cluster in the neighboring region of the P49 gene is involved in the transport of P49 to EMVs (Kamasaka et al., 2020). This gene cluster contained genes encoding a non-canonical type II secretion system (T2SS), homologs of surface polysaccharide-chain synthesis proteins, including a homolog of a flippase involved in bacterial extracellular polysaccharide synthesis, Wzx, and other proteins of unknown function (Figure 1; Supplementary Table 1). Gene disruption analysis indicated that P49 accumulates in the cells in the absence of the non-canonical T2SS, suggesting that this machinery transports P49 to the cell surface or extracellular space (Kamasaka et al., 2020). It was also found that P49 is secreted to the extracellular space without being loaded onto EMVs in the absence of *hm3343*, coding for Wzx, and other genes in the vicinity of the P49 gene, *hm3342*, *hm3344*, and *hm3345*, suggesting that these genes are essential for the synthesis of surface components of EMVs required for P49 loading onto EMVs (Kamasaka et al., 2020). However, the validity of this speculation and the identity of the EMV surface molecule required for P49 loading remain unclear.

The cell surfaces of Gram-negative bacteria are frequently covered with polysaccharide chains, such as capsular polysaccharide (CPS) and the O-antigen of lipopolysaccharide (LPS). Many of them have high antigenic activity and can be involved in cell defense and interaction with their environment (Comstock and Kasper, 2006). Since the surface of EMVs is assumed to mimic the structure of the cell surface, the surface polysaccharide of EMVs is also supposed to play important roles in these processes. Thus, to understand the physiological function of EMVs and to develop their application, it is important to clarify their surface polysaccharide structure. Previous studies determined the chemical structures of LPS and CPS of *S. vesiculosa* HM13. They showed that the LPS of this bacterium is a lipooligosaccharide (LOS) lacking an O-antigen (Di Guida et al., 2020) and that EMVs and cells are covered with the same CPS containing a novel aminosugar, named Shewanosamine (Casillo et al., 2022).

Wzx family proteins are generally involved in the biosynthesis of bacterial cell surface polysaccharides, i.e., CPS and O-antigen of LPS, by mediating the translocation of their oligosaccharide precursors from the cytoplasmic face to the periplasmic face of the inner membrane (Islam and Lam, 2013). Because *S. vesiculosa* HM13 produces CPS but not the O-antigen of LPS (Di Guida et al., 2020; Casillo et al., 2022; Casillo et al., 2019), we speculated that the Wzx homolog encoded by *hm3343* is involved in the synthesis of CPS.

In the present study, we investigated the function of HM3343, a Wzx homolog, and other proteins encoded by the genes near the P49 gene in the biosynthesis of the surface polysaccharide of *S. vesiculosa* HM13 and in P49 loading onto EMVs. We found that P49 is loaded onto EMVs through its interaction with CPS. This finding provides new insight into protein cargo loading onto bacterial EMVs and provides a molecular basis for developing a potential platform to display heterologous proteins on EMVs using P49 as a carrier.

## 2 Materials and methods

### 2.1 Bacterial strains and growth conditions

The strains used in this study are listed in Supplementary Table 2. The strains of *S. vesiculosa* HM13 were cultured in 30 mL LB liquid medium [Luria-Bertani: 1% (w/v) Tryptone (Nacalai Tesque, Kyoto, Japan), 1% (w/v) sodium chloride, and 0.5% (w/v) Difco Yeast Extract (Thermo Fisher Scientific, Waltham, MA, USA); pH 7.0] at 18 °C in a Bio Shaker BR-43FL incubator (Taitec, Saitama, Japan) at 180 rpm up to OD_600_ = 2–3. *Escherichia coli* S17-1/λ*pir* (Simon et al., 1983) used as a plasmid donor cell for the *pir*-dependent gene knockout plasmid, pKKPH, was grown aerobically in 5 mL LB medium at 37 °C. When required, uracil, 5-FOA, kanamycin (Km), rifampicin (Rif), and chloramphenicol (Cm) were added to the medium at final concentrations of 40 μg/mL, 1 mg/mL, 50 μg/mL, 50 μg/mL, and 30 μg/mL, respectively.

### 2.2 Gene deletion and complementation in *Shewanella vesiculosa* HM13

We obtained the gene-deleted and -inserted mutants based on a previously described method using the gene coding for orotidine-5′-phosphate decarboxylase (*pyrF*) (Ito et al., 2016). Based on the whole-genome sequence, we identified a *pyrF*-coding gene for *S. vesiculosa* HM13 (*pyrF*^HM13^). The construction method of the *pyrF*^HM13^-deleted strain (∆*pyrF*^HM13^) (Supplementary Table 2) and pKKPH plasmid for *pyrF*-based gene knockout is described in Supplementary procedures. ∆*p49*∆*pyrF*^HM13^ and ∆*hm3343*∆*p49*∆*pyrF*^HM13^ were constructed using the following two-step selection. The primers used are listed in Supplementary Table 3. The upstream and downstream regions of the P49 gene and those of *hm3343* were amplified from the genomic DNA of *S. vesiculosa* HM13 by PCR with the following primer pairs: *p49*_upfwd/*p49*_uprev, *p49*_downfwd/*p49*_downrev, *wzx*_upfwd/*wzx*_uprev, and *wzx*_downfwd/*wzx*_downrev, respectively. A linear fragment of the knockout plasmid pKKPH was amplified using pKKPH-invfwd and pKKPH-invrev. To generate the *pyrF*^HM13^-based gene-deletion plasmids (pKKPH-*p49* and pKKPH-*hm3343*), each of the upstream and downstream fragments was ligated with the pKKPH fragment using the NEBuilder HiFi DNA Assembly Cloning Kit (New England Biolabs, Japan, Tokyo, Japan). To insert pKKPH-*p49* into the genome of ∆*pyrF*^HM13^, pKKPH-*p49* was introduced into *E. coli* S17-1/λ*pir* and transferred to ∆*pyrF*^HM13^ by conjugation. Transformants were selected on LB plates containing uracil, Rif, and Km as the first selection. The selected mutant cells were then transferred to LB plates containing uracil, Rif, and 5-FOA to isolate 5-FOA-resistant single colonies as the second selection. The *p49*-deleted mutants were selected by colony PCR using the following set of primers: Check_*p49*fwd/Check_*p49*rev. To obtain a double mutant lacking both *p49* and *wzx*, pKKPH-*hm3343* was introduced into ∆*p49*∆*pyrF*^HM13^, and the *hm3343*-deleted mutant (∆*hm3343*∆*p49*∆*pyrF*^HM13^) was selected via two-step selection, as described above. The deletion of *hm3343* was confirmed by PCR using Check_*wzx*fwd/Check_*wzx*rev.

For *hm3343*-complementation, an expression plasmid carrying *hm3343* (p*hm3343*) was constructed. Linearized pJRD-Cm^r^ was amplified by PCR using the following primer set: pJRD-invfwd/pJRD-invrev. The DNA fragment of *hm3343* and the 474 bp upstream flanking region of *hm3343* containing its predicted promoter was amplified with the primer set *wzx*up500fwd/*wzx*rev. PCR products were assembled using the NEBuilder HiFi DNA Assembly Cloning Kit to obtain p*hm3343*. The *wzx* insertion was confirmed by DNA sequencing of the plasmid using the primers pJRD_CheckFW1, *wzx*_CheckFw2, and pJRD_CheckRV. The constructed plasmid was introduced into *E. coli* S17-1/λ*pir* cells and transferred to ∆*hm3343*∆*p49*∆*pyrF*^HM13^ and ∆*hm3343* by conjugation. ∆*hm3343*∆*p49*∆*pyrF*^HM13^ and ∆*hm3343* harboring p*hm3343* (∆*hm3343*∆*p49*∆*pyrF*^HM13^/p*hm3343* and ∆*hm3343*/p*hm3343*, respectively) were selected on LB plates containing Rif and Cm. As controls, empty pJRD-Cm^r^ was introduced into ∆*p49*∆*pyrF*^HM13^ and ∆*hm3343*∆*p49*∆*pyrF*^HM13^ to generate ∆*p49*∆*pyrF*^HM13^/p and ∆*hm3343*∆*p49*∆*pyrF*^HM13^/p, respectively.

### 2.3 Isolation of EMVs

EMVs were isolated using a previously reported method (Kamasaka et al., 2020; Chutkan et al., 2013). Cells were cultured in 30 mL of LB medium at 18 °C and harvested at the stationary phase (OD_600_ = 2.0) by centrifugation at 6,000 × *g* at 4 °C for 15 min with ALLEGRA X-30R (Beckman Coulter, Brea, CA, USA). The supernatant was centrifuged at 15,000 × *g* at 4 °C for 15 min with Sorvall LYNX4000 (Thermo Scientific, Waltham, MA, USA). To remove the remaining bacterial cells, the supernatant was filtered through a 0.45-μm pore polyethersulfone (PES) membrane filter. The filtrate was ultracentrifuged at 100,000 × *g* at 4 °C for 2 h using an Optima XE-90 (Beckman Coulter) to collect EMVs. The EMV pellets were suspended in 500 μL of Dulbecco’s phosphate buffered saline (135.9 mM NaCl, 2.7 mM KCl, 8.9 mM Na_2_HPO_4_, and 1.5 mM KH_2_PO_4_, pH7.2) supplemented with 0.2 M NaCl (DPBSS). To quantify the EMVs, the collected EMVs were stained with a lipophilic fluorescent molecule, *N*-(3-triethylammoniumpropyl)-4-(*p*-diethylaminophenyl-hexatrienyl) pyridinium dibromide (FM4-64) (Thermo Fisher Scientific, Waltham, MA, USA) as previously described (Kamasaka et al., 2020). EMV amounts were normalized based on FM4-64 fluorescence as a measure of total lipid content, ensuring equal membrane loading across samples.

### 2.4 Purification of P49

P49 for *in vitro* binding with EMVs was prepared from a 200 mL culture supernatant of ∆*hm3345*, which secretes P49 into the extracellular space without association with EMVs. Bacterial cells were removed by centrifugation at 6,000 × *g* at 4 °C for 15 min with ALLEGRA X-30R. After removing the cells, the supernatant was concentrated with an Amicon Ultra filter (Merck Millipore, Billerica, MA, USA; 30 kDa molecular weight cut-off) and subjected to ultracentrifugation at 100,000 × *g* for 2 h at 4 °C to remove EMVs. P49 in the supernatant (post-vesicle fraction, PVF) was then purified with Superose 12 10/300 GL column (GE Healthcare, Little Chalfont, UK) (flow rate 0.4 mL/min, fraction volume 2.0 mL). Fractions were eluted with PBS buffer (137 mM NaCl, 2.7 mM KCl, 10 mM Na_2_HPO_4_·12H_2_O, and 1.8 mM KH_2_PO_4_, pH 7.4). The purity of P49 in the eluted fractions was analyzed by SDS-PAGE and staining with Coomassie Brilliant Blue G-250. The concentration of P49 was measured using a BCA protein assay kit (Nacalai Tesque) with bovine serum albumin as a standard.

### 2.5 Extraction and characterization of CPS

CPS was extracted from the EMVs using a previously reported method (Tipton and Rather, 2019) with some modifications. Briefly, 200 μL of CPS extraction buffer (60 mM Tris–HCl (pH 8.0), 0.045 mM CaCl_2_, and 21 mM MgCl_2_) was added to 100 μL of the EMV suspension, which contained EMVs corresponding to 5 mL culture. The mixture was subjected to three freeze–thaw cycles at −80 °C and 37 °C and treated with 5 units of DNase I (TaKaRa Bio, Otsu, Japan) and 20 ng RNase (Nippon Gene, Tokyo, Japan) at 37 °C for 30 min to remove nucleic acids. The reaction was stopped by the addition of SDS to a final concentration of 0.33% (w/v) and boiling. The mixture was then treated with 300 μg proteinase K (Thermo Scientific) at 60 °C for 1 h to remove proteins and centrifuged to remove debris. Finally, CPS was precipitated overnight in 75% ice-cold ethanol. The CPS pellet was dissolved in SDS-PAGE loading buffer (125 mM Tris–HCl (pH 6.8), 10% (v/v) 2-mercaptoethanol, 4% (w/v) SDS, 10% (w/v) sucrose, and 0.01% (w/v) bromophenol blue) and heated at 100 °C for 5 min. CPS was subjected to 12.5% SDS-PAGE and stained with Alcian Blue Solution (Wako Pure Chemical Industries, Osaka, Japan).

To measure the weight of CPS, the cells and EMVs of the rifampicin-resistant mutant of *S. vesiculosa* HM13 (HM13-Rif^r^) and its *wzx*-disrupted mutant (∆*hm3343*) were extracted as already reported (Casillo et al., 2022). The extracts were hydrolyzed under mild acid conditions, and CPS was purified by gel filtration chromatography. We measured the weight of CPS, dried cells, and dried EMVs, and calculated the weight percentage of CPS as follows: [purified CPS (mg) /dried material (mg)] × 100.

### 2.6 *In vitro* P49 binding assay

Purified P49 was mixed with an equal volume of EMV suspension (100 μL each; equivalent to the amount of EMVs isolated from 5 mL of culture) to achieve final P49 concentrations of 0.375, 0.75, 1.5, or 2.0 μM. The mixtures were incubated at 4 °C for 1 h or 15 h, as indicated in the Results section. After incubation, the P49-EMV mixtures were ultracentrifuged at 100,000 × *g* for 2 h at 4 °C using an Optima XE-90 to collect the EMV pellet and supernatant. The resulting pellet was resuspended in 500 μL of PBS. Both the resuspended pellet and the supernatant were then subjected to trichloroacetic acid (TCA) precipitation to concentrate the proteins. Specifically, TCA was added to each sample to a final concentration of 10% (w/v). After incubation on ice for 30 min, the mixtures were centrifuged at 15,000 × *g* for 15 min at 4 °C. The obtained precipitates were washed twice with acetone prechilled at −30 °C, air-dried, and then redissolved in 40 μL of SDS-PAGE loading buffer. After heating at 95 °C for 5 min, the samples were subjected to 12.5% SDS-PAGE followed by western blot analysis.

For western blot analysis, proteins were transferred onto a PVDF membrane, Immobilon-P (Millipore). After blocking with 5% (w/v) skim milk, the membrane was incubated with a rabbit polyclonal anti-P49 antibody raised against the C-terminal peptide (ASDRGFVSNTSNGS), as described previously (Chen et al., 2019). Subsequently, the membrane was incubated with a secondary antibody, Goat Anti-Rabbit IgG (H + L)-HRP Conjugate (Bio-Rad Laboratories, Hercules, CA, USA). P49-specific protein bands were detected using the Chemilumi-One Super kit (Nacalai Tesque), and the signals were visualized with a C-Digit Blot Scanner (LI-COR Biosciences, Lincoln, NE, USA).

### 2.7 TEM analysis

The TEM images of the EMVs were obtained as follows. The EMV samples (2.5 μL) applied to hydrophilized carbon-coated copper grids were treated twice with 0.5% uranyl acetate. A JEM-1400 transmission electron microscope (JEOL, Tokyo, Japan) at an accelerating voltage of 120 kV and a charge-coupled device (CCD) camera (built-in camera within JEM-1400) were used to obtain images. The diameters of EMVs and peripheral particles around EMVs were measured using the image analysis software Fiji (ImageJ2 version 2.3.0/1.53f) (Schindelin et al., 2012).

### 2.8 CD spectroscopy

CD spectra of EMV-bound and free P49 were obtained using a J-820 CD spectrometer (JASCO, Tokyo, Japan) with a 1-mm path length quartz cuvette. Purified P49 (0.3 mg/mL) was mixed with an equal volume (150 μL) of EMV suspension corresponding to EMVs isolated from 2.5 mL culture and incubated at 4 °C for 15 h, resulting in a final P49 concentration of 0.15 mg/mL. Far-UV spectra were recorded from 200 to 250 nm at 18 °C. As a background control, spectra of EMVs incubated with buffer lacking P49 were measured under identical conditions and subtracted from the EMV-containing sample spectra to ensure that the observed signals were solely attributable to P49.

### 2.9 Prediction of protein localization

Protein localization was predicted using PSORTb version 3.0.3.^1^

## 3 Results

### 3.1 Wzx-dependent synthesis of EMV-associated CPS

**Figure 2:**
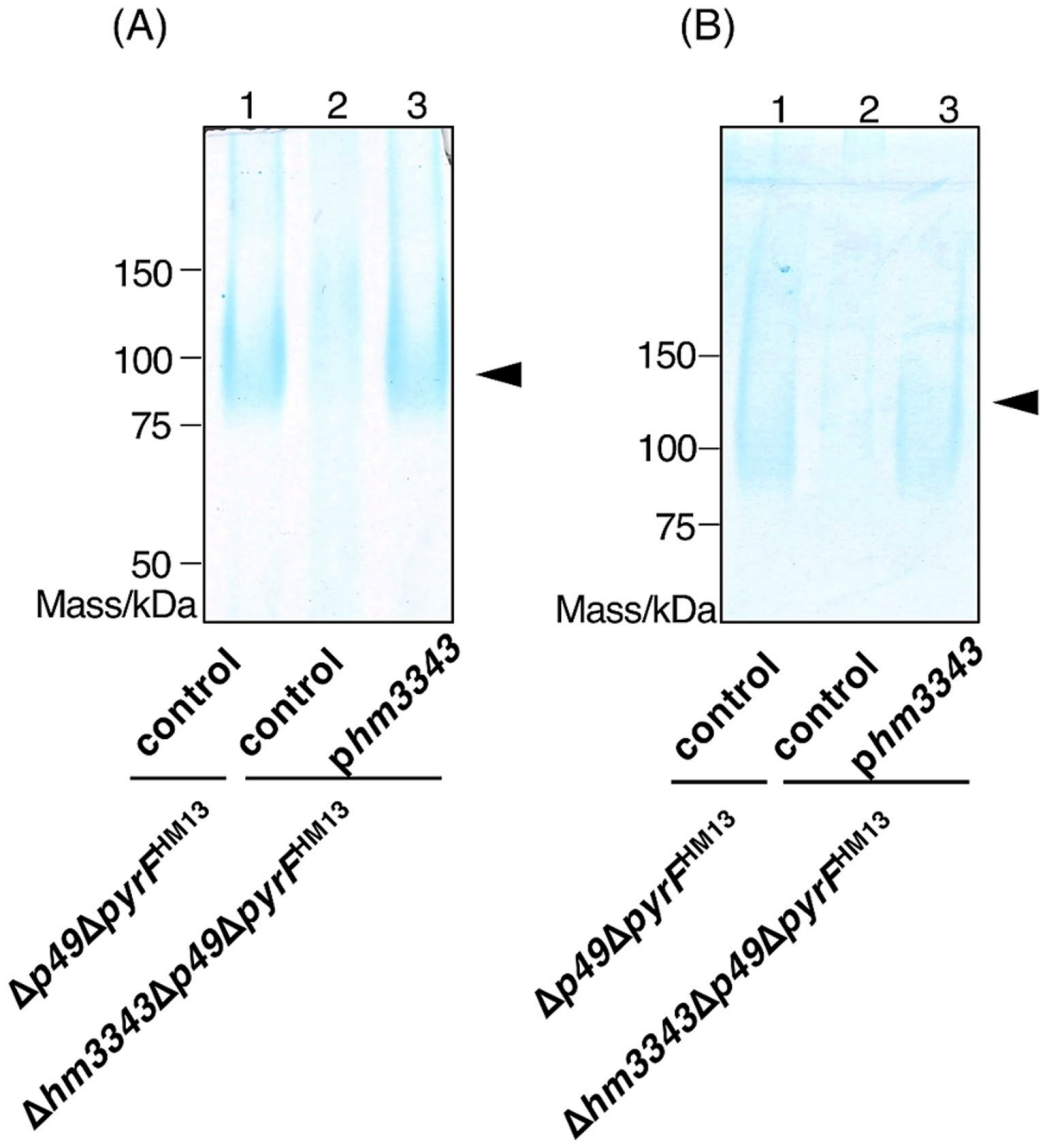
CPS extracted from EMVs and cells of *Shewanella vesiculosa* HM13. Alcian blue-stained SDS-PAGE analysis of CPS extracted from EMVs **(A)** and cells **(B)** of ∆*p49*∆*pyrF*^HM13^ harboring an empty plasmid, pJRD-Cm^r^, (control) and its *hm3343*-deletion mutant, ∆*hm3343*∆*p49*∆*pyrF*^HM13^, harboring pJRD-Cm^r^ (control) or the *hm3343*-expression plasmid (p*hm3343*). The arrowheads indicate the positions of CPS. Cropped from original gels; the full-length images are shown in Supplementary Figure 1.

EMVs of the *hm3343*-deleted mutant (∆*hm3343*∆*p49*∆*pyrF*^HM13^) and its parent strain (∆*p49*∆*pyrF*^HM13^) (Supplementary Table 2) were subjected to CPS analysis following the Tipton and Rather method (Tipton and Rather, 2019). In this study, we used a *pyrF*-deleted mutant of *S. vesiculosa* HM13 as the parental strain, in which the *pyrF* gene encoding orotidine-5′-phosphate decarboxylase was deleted. This uracil auxotrophic mutant enables efficient markerless gene deletion and insertion using the 5-fluoroorotic acid (5-FOA) selection system. In addition, the *p49*-deleted background was used to prevent the confounding effects of endogenous P49 in subsequent binding assays, where the CPS-dependent association of purified P49 with EMVs was investigated. For the CPS extract obtained from EMVs of the parent strain, a smeared band of CPS was detected in the Alcian Blue-stained SDS-PAGE gel, whereas this major band was barely visible for the *hm3343*-deleted mutant (Figure 2A). The CPS band was detected for the EMV extract from ∆*hm3343*∆*p49*∆*pyrF*^HM13^ harboring the *hm3343* expression plasmid (p*hm3343*) (Figure 2A) as well as for the cells of ∆*p49*∆*pyrF*^HM13^ and ∆*hm3343*∆*p49*∆*pyrF*^HM13^ harboring p*hm3343*, but not for ∆*hm3343*∆*p49*∆*pyrF*^HM13^ cells (Figure 2B). We also determined the yield of CPS extracted and purified from cells and EMVs. In dried cells, the weight percentage of CPS was 1.3% in the parent strain (HM13-Rif^r^) and 0.065% in ∆*hm3343*, while in dried EMVs, it was 4.9 and 1.2%, respectively. These results indicate that the Wzx homolog, encoded by *hm3343* in the vicinity of the P49 gene, is involved in the cellular synthesis of CPS that is associated not only with cells but also with EMVs.

### 3.2 CPS-dependent P49 binding to EMVs

**Figure 3:**
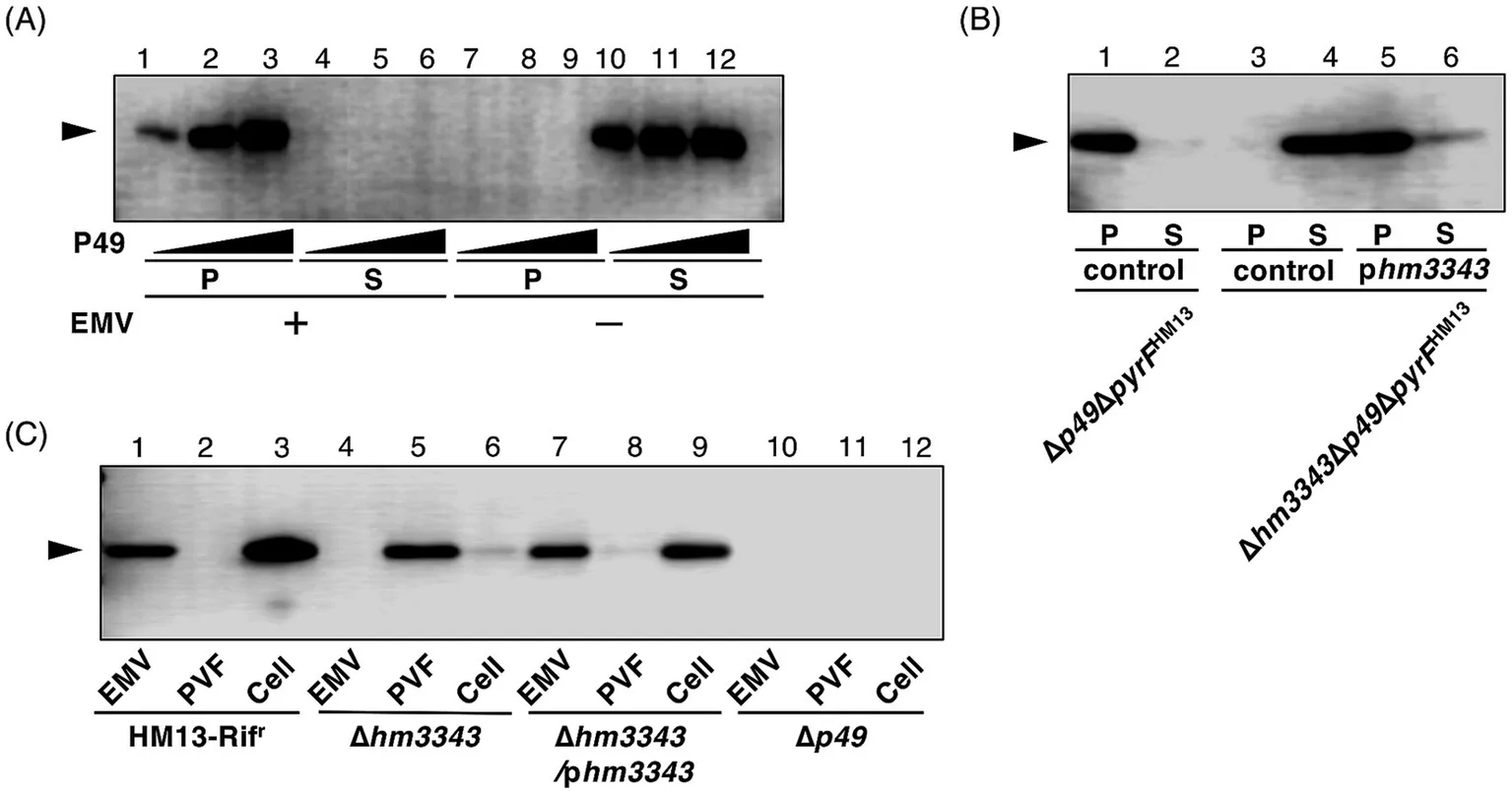
*In vitro* and *in vivo* P49 binding to EMVs. **(A)** *In vitro* binding of P49 to P49-free EMVs of ∆*p49*∆*pyrF*^HM13^. After incubation, samples were fractionated by ultracentrifugation, and the supernatant and pellet fractions were subjected to western blotting with an antibody against P49. As a control, purified P49 without EMVs was subjected to the analysis. P and S indicate pellet and supernatant fractions, respectively. **(B)** *In vitro* P49 binding to EMVs from ∆*p49*∆*pyrF*^HM13^ harboring an empty plasmid, pJRD-Cm^r^, (control) and *hm3343*-deleted mutant, ∆*hm3343*∆*p49*∆*pyrF*^HM13^, harboring pJRD-Cm^r^ (control) or the expression plasmid of *hm3343* (p*hm3343*). P and S indicate pellet and supernatant fractions, respectively. **(C)** *In vivo* localization of P49 was analyzed for the parent (HM13-Rif^r^), ∆*hm3343*, and ∆*hm3343* harboring the *hm3343*-expression plasmid (∆*hm3343*/p*hm3343*). P49 in the EMVs, PVF, and cells was detected by western blotting with an antibody against P49. The amount of each sample corresponded to that obtained from 50 μL of culture. The position of P49 is indicated by the arrowhead. These results were confirmed in three independent experiments. Cropped from original blots; full-length versions of the blots corresponding to panels A–C are shown in Supplementary Figure 3.

To investigate P49 loading onto EMVs *in vitro*, we conducted a P49-binding assay using purified P49 (Supplementary Figure 2) and P49-free EMVs prepared from a mutant lacking P49 (∆*p49*). P49 was purified from the post-vesicle fraction (PVF), an EMV-free culture supernatant obtained by pelleting EMVs, from a mutant that lacks *hm3345* encoding glycerophosphodiester phosphodiesterase homolog (GdpD) (DDBJ: LC431025) (∆*hm3345*), in which P49 is released into PVF without being associated with EMVs (Kamasaka et al., 2020). The *in vitro* binding assay was conducted by mixing 100 μL of different concentrations of purified P49 with an equal volume of EMVs (equivalent to the amount in 5 mL culture) to final concentrations of 1.5, 0.75, and 0.375 μM, respectively, and incubating for 15 h. As shown in Figure 3A, purified P49 was observed in the pellet fraction in the presence of EMVs, and the amount of precipitated P49 increased in accordance with increased input P49. In contrast, in the absence of EMVs, the P49 band was detected only in the supernatant. These results indicate that P49 was associated with EMVs *in vitro* and co-precipitated with EMVs.

Next, to investigate the effect of the lack of CPS on the interaction between P49 and EMVs, we conducted the *in vitro* binding assay using P49 and EMVs from the CPS-less mutant (∆*hm3343*∆*p49* ∆*pyrF*^HM13^). With a final P49 concentration of 2 μM, the protein was recovered in the supernatant fraction (Figure 3B). The ability of EMVs to interact with P49 was restored by introducing an *hm3343*-complementation plasmid, p*hm3343* (∆*hm3343*∆*p49*∆*pyrF*^HM13^/p*hm3343*). The dissociation of P49 from EMVs was also observed *in vivo* for ∆*hm3343* (Figure 3C). The P49-loading capacity of the EMVs of this strain was restored by the introduction of p*hm3343* (∆*hm3343*/p*hm3343*) (Figure 3C). Since HM3343 is an inner membrane protein, it is unlikely to be directly involved in the binding of externally added P49 to EMVs. Therefore, these results strongly suggest that P49 binds to EMVs via an interaction with the CPS synthesized in an HM3343-dependent manner.

**Figure 4:**
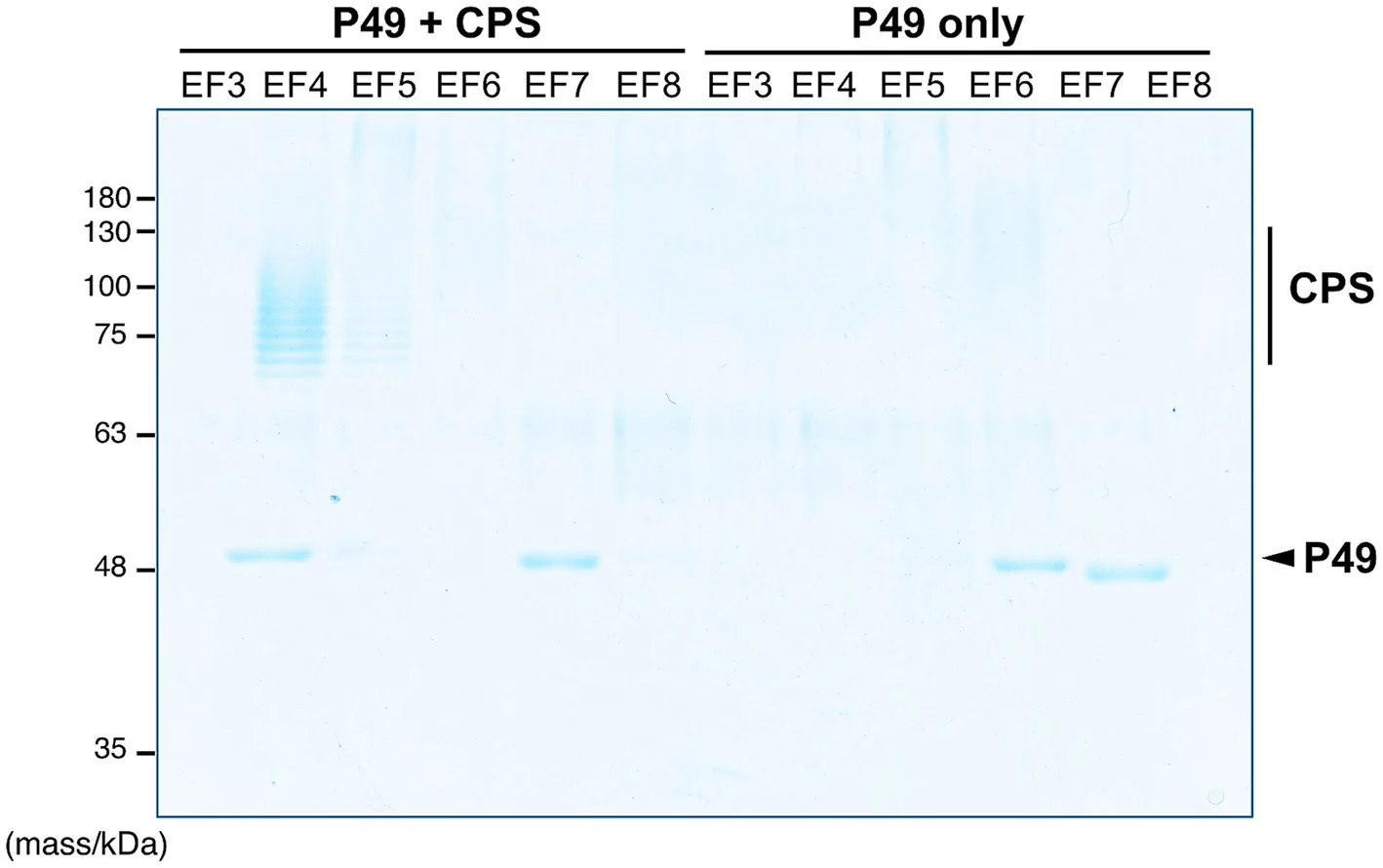
*In vitro* binding capacity of the purified P49 to CPS. SDS-PAGE analysis of the elution fractions of the gel-filtration chromatography. The elution fractions 3–8 (EF3–EF8) were subjected to SDS-PAGE, and the gel was stained with Alcian Blue and CBB G-250. The positions of CPS and P49 are shown in the figure.

We further investigated the direct interaction between P49 and CPS through *in vitro* binding and subsequent gel filtration chromatography. In the presence of CPS, P49 was eluted as a high-molecular-weight complex (Figure 4), indicating that P49 has a binding capacity with CPS. To evaluate the specificity of the P49-CPS interaction, we performed a binding assay using bovine serum albumin (BSA) as a control. SDS-PAGE analysis showed that a faint BSA band was detected in the CPS-containing fractions; however, a much stronger band was observed in low-molecular-weight fractions distinct from the CPS-containing fractions (Supplementary Figure 4). Therefore, the CPS of this strain exhibits much higher binding affinity for P49 than for BSA. These results collectively established that CPS synthesized by the HM3343-dependent pathway plays a crucial role in the association between P49 and EMVs both *in vivo* and *in vitro*.

### 3.3 Occurrence of P49 as peripheral particles around EMVs

**Figure 5:**
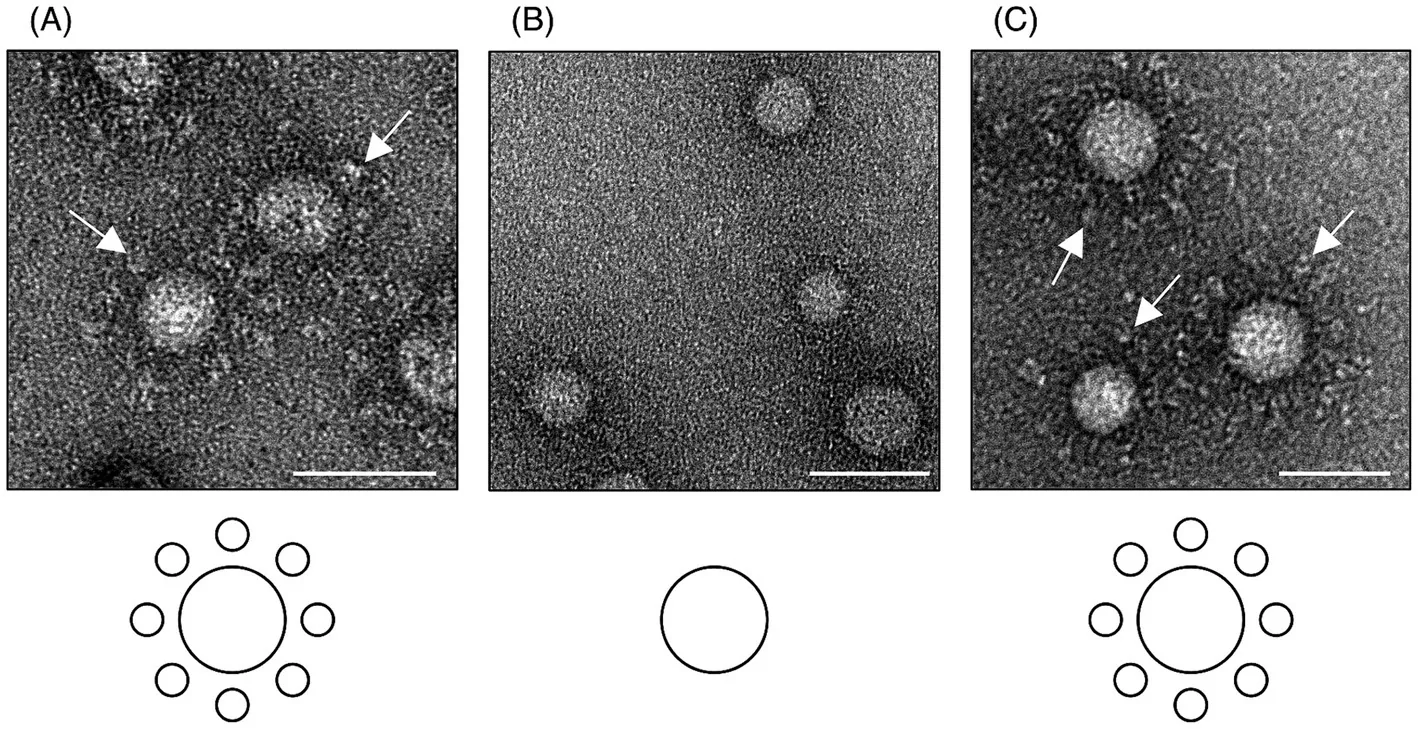
TEM analysis of EMVs with and without loaded P49. TEM images of EMVs from ∆*pyrF*^HM13^ **(A)** and ∆*p49*∆*pyrF*^HM13^ **(B)** and EMVs from ∆*p49*∆*pyrF*^HM13^ incubated with purified P49 *in vitro* **(C)**. The arrows indicate the peripheral particles. For clarity, only some of them are marked with arrows. Schematic diagrams of EMVs and peripheral particles are shown below their respective images. The diameters of EMVs and peripheral particles were measured using Fiji (ImageJ2) software. Scale bars indicate 50 nm.

We analyzed the morphology of EMVs from the parent strain (∆*pyrF*^HM13^) and ∆*p49*∆*pyrF*^HM13^ and EMVs from ∆*p49*∆*pyrF*^HM13^ incubated with P49. Transmission electron microscopy (TEM) analysis of the parent EMVs demonstrated the presence of peripheral particles with a diameter of 10.2 +/− 3.0 nm (*n* = 100) on the surface of spherical vesicles with a diameter of 35.0 +/− 3.2 nm (*n* = 100) (Figure 5A). Peripheral particles were not observed around the EMVs of the mutant lacking P49 (Figure 5B). Peripheral particles with a size of 9.5 +/− 3.0 nm (*n* = 100), similar to those observed for the EMVs of the parent strain, were observed around the EMVs after *in vitro* binding of purified P49 (Figure 5C). These results suggest that P49 constitutes peripheral particles of the EMVs.

### 3.4 Characterization of the *in vitro* association between P49 and EMVs

To further characterize the *in vitro* association between P49 and EMVs, we compared the secondary structure of EMV-free P49 with that of P49 associated with EMVs from ∆*p49* by circular dichroism (CD) analysis (Supplementary Figure 5). We observed that the purified P49 did not exhibit a typical CD signal of proteins with α-helix and β-sheet structures. In contrast, P49 incubated with EMVs from ∆*p49* showed a typical CD signal of β-sheet proteins, with the lowest negative ellipticity around 215 nm. The structural change in P49 caused by incubation with the EMVs from ∆*p49* was not detected when the EMVs of ∆*hm3343*, which did not bind to P49 as shown in Figure 3C, were used.

To examine whether divalent metal ions such as Ca^2 +^ and Mg^2+^ play a role in the interaction between P49 and EMVs, the cargo loading assay using P49 and EMVs from ∆*p49*∆*pyrF*^HM13^ was conducted in the presence of a chelating agent, ethylenediaminetetraacetic acid (EDTA). In the binding assay, the final concentration of P49 was 2 μM. As shown in Supplementary Figure 6, supplementation with 20 mM EDTA did not affect the association of P49 with EMVs, and no protein band was observed in the supernatant. Thus, it is unlikely that divalent metal ions are involved in the binding of P49 to EMVs.

We also analyzed the effects of ionic strength on the interaction between P49 and EMVs by increasing the NaCl concentration in the *in vitro* P49-loading assay mixture. We observed that P49 was co-precipitated with EMVs even in the presence of 1.0 M NaCl (Supplementary Figure 7), suggesting that electrostatic interactions do not play an important role in the interaction between P49 and EMVs.

### 3.5 Involvement of P49-neighboring genes in CPS synthesis and P49 loading onto EMVs

**Figure 6:**
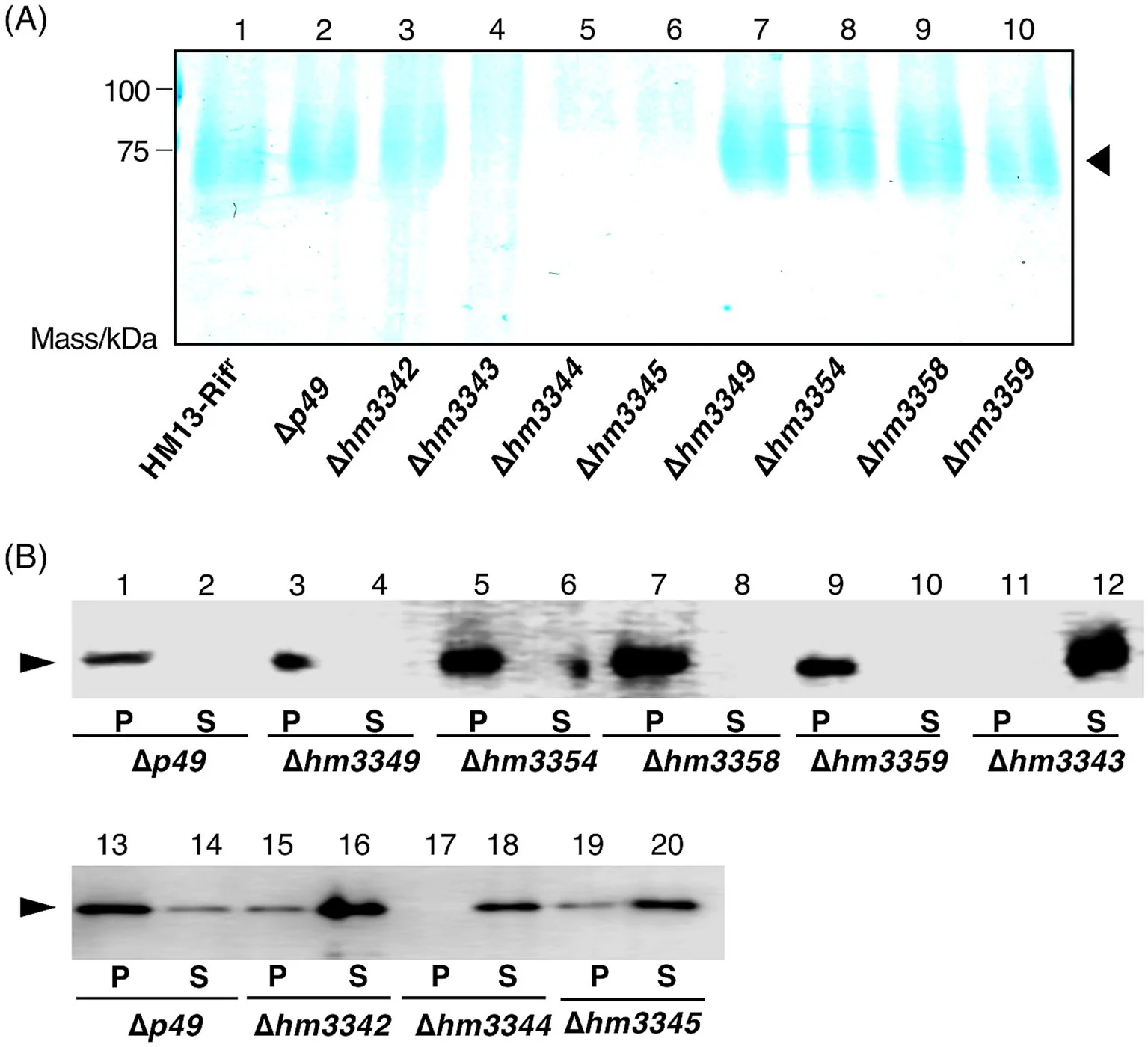
Effects of the disruption of the genes around the P49 gene on the CPS and the P49-binding capacity of the EMVs. **(A)** Alcian Blue-stained SDS-PAGE analysis of CPS extracted from EMVs prepared from the parent strain and gene-disrupted mutants. The arrowhead indicates the position of the broad CPS band. **(B)** *In vitro* P49-binding assay using EMVs from the parent and mutant strains. Purified P49 (final concentration, 2 μM) was incubated with EMVs from the parent and mutant strains for 1 h at 4 °C. After incubation, the samples were fractionated into pellet (P) and supernatant (S) by ultracentrifugation and analyzed by western blotting with an anti-P49 antibody. The arrowheads indicate the position of P49. Cropped from the original gel and blots; full-length versions of the gel and blots corresponding to the panels are shown in Supplementary Figure 8.

In the vicinity of the P49 gene, we identified genes encoding the subunits of a non-canonical T2SS (*hm3360* (*gspG2*, major pseudopilin), *hm3359* (*gspE2*, secretion ATPase), *hm3358* (*gspF2*, inner membrane platform), *hm3354* (*gspK2*, minor pseudopilin), *hm3351* (*gspB2*, accessory protein), and *hm3349* (*gspD2*, outer membrane conduit)) and genes encoding homologs of polysaccharide export protein (*hm3363*), undecaprenyl-phosphate alpha-*N*-acetylglucosaminyl 1-phosphate transferase (*hm3362* and *hm3361*), glycerophosphodiester phosphodiesterase homolog (*hm3345*), phosphoethanolamine transferase (*hm3344*), Wzx flippase (*hm3343*), nitroreductase (*hm3342*), and UDP-glucose 6-dehydrogenase (*hm3341*) (Chen et al., 2019; Figure 1; Supplementary Table 1). To examine whether these gene products are involved in CPS synthesis, EMVs from the mutants obtained by disrupting *hm3359*, *hm3358*, *hm3354*, *hm3349*, *hm3345*, *hm3344*, *hm3343*, and *hm3342* individually were subjected to CPS analysis. The broad band observed in the low-mobility area for the parent strain was barely visible for ∆*hm3345*, ∆*hm3344*, and ∆*hm3343* (Figure 6A). In contrast, the CPS band was detected in EMVs from the mutants that lacked the non-canonical T2SS components (∆*hm3359*, ∆*hm3358*, ∆*hm3354*, and ∆*hm3349*) and the nitroreductase homolog (∆*hm3342*). To examine whether P49 was loaded onto EMVs from these mutants, an *in vitro* P49-binding assay was conducted. EMVs from the mutants that lacked the non-canonical T2SS components showed the binding capacity for purified P49, whereas the disruption of *hm3345*, *hm3344*, *hm3343*, and *hm3342* impaired P49 loading onto EMVs (Figure 6B). These results demonstrated that P49 shows markedly low affinity for EMVs derived from CPS-deficient mutants, including those other than the *hm3343*-deficient strain.

## 4 Discussion

**Figure 7:**
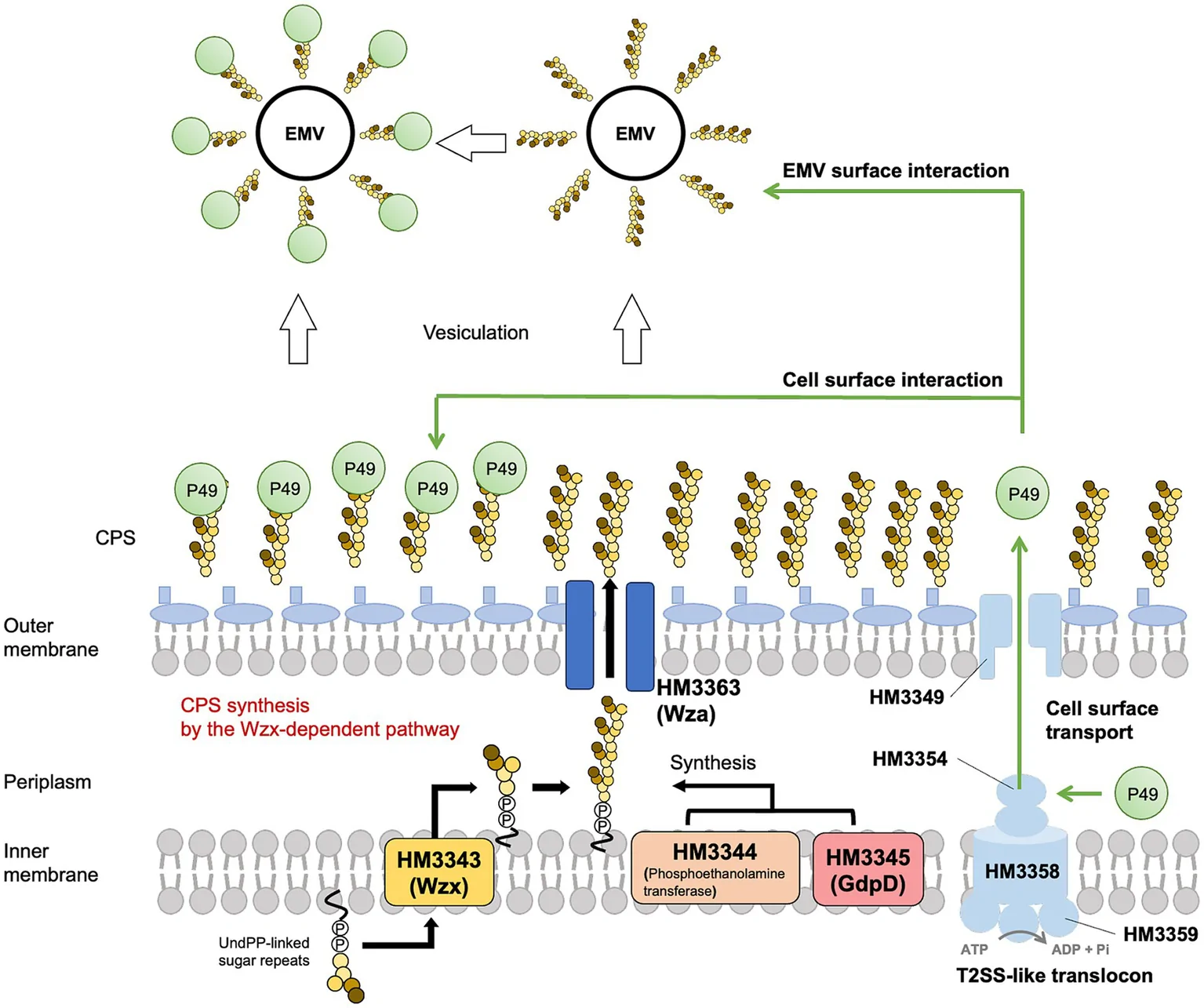
The model of P49 transport onto EMVs of *Shewanella vesiculosa* HM13. P49 is translocated across the outer membrane through the T2SS-like translocon and is subsequently loaded onto EMVs through its interaction with CPS. In CPS synthesis, Wzx encoded by *hm3343* mediates the translocation of undecaprenyl pyrophosphate (UndPP)-linked sugar repeats across the inner membrane from the cytoplasm to the periplasmic space. Outer membrane translocation of CPS is likely mediated by the Wza translocon encoded by *hm3363* (Yuan et al., 2026). Homologs of phosphoethanolamine transferase (HM3344) and glycerophosphodiester phosphodiesterase (GdpD) (HM3345) are also required for CPS synthesis. Protein localization was predicted using the PSORTb version 3.0.3 as described in the Materials and methods.

In this study, we identified the genes involved in the synthesis of the EMV-associated CPS of *S. vesiculosa* HM13, whose chemical structure was recently determined and found to be identical to that of the cellular CPS (Casillo et al., 2022) (Figures 2, 6A). By disrupting these genes, we revealed that P49, the major cargo protein of EMVs, is associated with EMVs in a CPS-dependent manner. To demonstrate the CPS-dependent association of P49 with EMVs, we conducted an *in vitro* P49-binding assay. Purified P49 bound to EMVs from the parent strain producing CPS, whereas it did not bind to EMVs from mutants that did not produce CPS (Figures 3B, 6B). We observed small particles around the EMVs from the parent strain and those from the P49-deficient mutant incubated with P49 *in vitro* by TEM analysis, whereas such particles were not observed around EMVs not carrying P49 (Figure 5). Direct interaction between P49 and CPS was also demonstrated by a gel-filtration-based *in vitro* binding assay (Figure 4). The specificity of this interaction was examined using BSA as a control. BSA showed a much smaller shift in its elution profile in the presence of CPS than P49 (Figure 4; Supplementary Figure 4). These results clearly indicate that P49 is loaded onto EMVs via its interaction with CPS on the surface of the EMVs (Figure 7). CD analysis suggested that P49 adopted a β-strand-rich structure upon its binding to EMVs (Supplementary Figure 5). Furthermore, the P49–EMV association was resistant to both high ionic strength (1.0 M NaCl) and a divalent metal chelator (EDTA) (Supplementary Figures 6, 7), suggesting that simple electrostatic interactions or divalent metal ions are not major determinants of this association. Considering the morphological size of the peripheral particles of EMVs, it is reasonable to assume that the particles consist of P49 oligomers or P49 complexed with CPS (Figure 5). Previous studies showed that P49 accumulates in the cells of gene-disrupted mutants that lack the T2SS-like machinery encoded by the genes upstream of the P49 gene (*hm3359*, *hm3358*, *hm3354*, and *hm3349*) (Kamasaka et al., 2020), indicating that this machinery is responsible for the translocation of P49 across the outer membrane (Figure 7).

We previously found that P49 is released into the culture supernatant without being loaded onto EMVs upon disruption of genes coding for homologs of Wzx (HM3343), phosphoethanolamine transferase (HM3344), and GdpD (HM3345), located downstream of the P49 gene (Kamasaka et al., 2020). However, it was unclear why P49 was not loaded onto EMVs in these mutants. In the present study, we revealed that the loss of CPS on the EMV surface is a major cause of the impaired P49 loading observed in these mutants. We found that HM3343, HM3344, and HM3345 are required for CPS production (Figures 2, 6A, 7). The primary structure of HM3343 strongly suggests that it functions as a flippase to translocate CPS precursors across the inner membrane (Islam and Lam, 2013; Chua et al., 2021). However, it is not clear how HM3344 and HM3345 are involved in CPS synthesis based solely on in silico analysis. Although HM3344 has sequence similarity to phosphoethanolamine transferase (Samantha and Vrielink, 2020; Huang et al., 2018), no phosphoethanolamine residue was found in CPS of this strain (Casillo et al., 2022). As to HM3345, most homologs of this protein are registered as hypothetical proteins in databases. Although some homologs are annotated as glycerophosphodiester phosphodiesterase homologs (Fan et al., 2001; Ohshima et al., 2008), their relevance to CPS synthesis has not been reported. Thus, their function in CPS synthesis should be examined experimentally in future studies. Notably, the disruption of *hm3342*, adjacent to *hm3343* (*wzx*), did not cause the loss of the CPS band in SDS-PAGE analysis (Figure 6A) but resulted in the loss of interaction between EMVs and P49 (Figure 6B). We recently analyzed CPS of the *hm3342*-disrupted mutant (∆*hm3342*) and found that, in addition to the CPS found in its parental strain, this mutant produces an additional CPS with a repeating disaccharide unit consisting of a 4-substituted glucose alternating with a 3-substituted *N*-acetylglucosamine (Casillo et al., 2024). This result raises the possibility that the presence of two distinct CPS species inhibits the interaction between P49 and EMVs. In addition to *hm3343*, *hm3344*, *hm3345*, and *hm3342*, many genes in the vicinity of the P49 gene are speculated to be involved in CPS biosynthesis, such as *hm3341* encoding Ugd, *hm3361* and *hm3362* encoding WecA, and *hm3363* encoding Wza (Figure 1, Supplementary Table 1). Because the CPS of *S. vesiculosa* HM13 has a novel structure with a unique amino sugar, which has never been reported before (Casillo et al., 2022), CPS biosynthetic enzymes with unique catalytic properties are expected to be discovered.

The finding that CPS contributes to P49 association with EMVs suggests a previously unrecognized role of CPS in cargo protein loading. Previous studies have shown that proteins can be enriched in EMVs through membrane-associated mechanisms, including anchoring to anionic LPS in *Porphyromonas gingivalis* (Haurat et al., 2011; Veith et al., 2014) and enrichment of lipoproteins with N-terminal acyl groups in *Bacteroides* (Elhenawy et al., 2014; Valguarnera et al., 2018). In contrast, the present study supports a model in which a major cargo protein is associated with EMVs through interaction with CPS on the vesicle surface. To our knowledge, this represents a distinct mode of cargo protein association with bacterial EMVs.

This P49–CPS system may also provide a useful starting point for future development of EMV surface engineering strategies. Recombinant protein display on EMVs has been explored for the development of nanoscale materials, including vaccines and therapeutic agents (Cheng et al., 2021; Daleke-Schermerhorn et al., 2014; van den Berg van Saparoea et al., 2018). Currently available approaches for displaying foreign proteins on EMVs often rely on membrane anchor proteins as fusion or binding partners (van den Berg van Saparoea et al., 2018; Long et al., 2022). Because CPS is located outside the membrane, CPS-mediated association may offer an alternative strategy for post-vesiculation protein loading onto isolated EMVs. Consistent with this possibility, externally added P49 bound to isolated P49-deficient EMVs *in vitro*. However, further studies will be required to establish this system as a practical protein display platform, including identification of P49 regions responsible for CPS binding, determination of the CPS structural features required for the interaction, quantitative analysis of binding affinity and capacity, and demonstration of functional loading of heterologous proteins.

Although establishing a system for foreign protein display on EMVs using CPS as a scaffold remains an attractive target for future research, this study is significant in that it provides evidence for a new mode of protein loading onto EMVs and highlights the potential of using CPS as a binding scaffold. In particular, this strategy provides a promising framework for applications such as EMV-based vaccines, targeted drug delivery, and functional biomaterials. The detailed mechanism underlying the interaction between P49 and CPS remains to be fully elucidated in future research. Determination of the structural moieties of P49 and CPS involved in their binding will be important for developing the protein-loading system described above. Importantly, the *in vitro* cargo-loading system established in this study will facilitate such mechanistic analyses.

## Data Availability

The raw data supporting the conclusions of this article will be made available by the authors, without undue reservation.

## Footnotes

1
https://www.psort.org/psortb/
