# Supplementary material for: Capsular polysaccharide-mediated protein loading onto extracellular membrane vesicles of Shewanella vesiculosa HM13
Source: Front Microbiol. 2026 Sep 9;17:1934777. doi: 10.3389/fmicb.2026.1934777 (PMC13597727; doi:10.3389/fmicb.2026.1934777)
Supplement: Supplementary file 1 [file Data_Sheet_1.pdf]

Supplementary Material

Capsular polysaccharide-mediated protein loading onto extracellular membrane vesicles of *Shewanella vesiculosa* HM13

Kouhei Kamasaka[1], Jun Kawamoto[1*], Taiku Tsudzuki[1], Yuanzheng Yang[1], Yuying Liu[1], Tomoya Imai[2], Angela Casillo[3], Takuya Ogawa[1], Maria Michela Corsaro[3], Tatsuo Kurihara[1*]
*Corresponding authors: Tatsuo Kurihara; Jun Kawamoto
Email: kurihara@scl.kyoto-u.ac.jp; kawamoto.jun.4s@kyoto-u.ac.jp

This PDF file includes:
Supplementary procedures
Supplementary Tables 1-3
Supplementary Figures 1-8

Supplementary procedures

Construction of a *pyrF*-based gene knockout plasmid and preparation of Δ*pyrF*^{HM13}

A DNA fragment composed of the upstream and downstream flanking regions (1,590 and 1,447 bp, respectively) of *pyrF*^{HM13} was introduced into the SmaI site of pKNOCK-Km^r to obtain pK*pyrF*^{HM13}. The upstream and downstream flanking regions of *pyrF*^{HM13} and linearized pKNOCK-Km^r were amplified using the following primer sets: *pyrF*^{HM13}+1500-fw/*pyrF*^{HM13}-del2, *pyrF*^{HM13}-del1/*pyrF*^{HM13}+1500-rv, and pKKPH-invfwd/pKKPH-invrev, respectively (Supplementary Table 3). DNA fragments of the flanking regions and linearized plasmid were ligated using the NEBuilder HiFi DNA Assembly Cloning System kit (New England Biolabs, Japan, Tokyo, Japan). The resulting plasmid, pK*pyrF*^{HM13}, was introduced into *E. coli* S17-1/λ*pir* and then transferred to *S. vesiculosa* HM13-Rif^r by conjugation as previously described[1]. Transformants grown on LB agar plates containing kanamycin (Km) and rifampicin (Rif) were selected. Single colonies were picked up, and plasmid integration into the genome was confirmed by diagnostic PCR with the primer set *pyrF*^{HM13}-1.7up-fw and pKNOCK-0.15dw-rv. The colony harboring the integrated plasmid was cultured in 5 mL of LB media at 18 °C until the OD_{600} reached approximately 1.0, and then the cells were spread on LB plates containing 40 µg/mL uracil, 50 µg/mL Rif, and 1 mg/mL 5-fluoroorotic acid (5-FOA). After two rounds of selection with 5-FOA, the deletion of *pyrF*^{HM13} from the genome was confirmed by diagnostic PCR using the following primer set: Check_*pyrF*^{HM13}fwd and Check_*pyrF*^{HM13}rev.

Construction of a gene-deletion plasmid, pKKPH

A *pyrF*^{HM13}-based gene-knockout plasmid, pKKPH, was constructed. The *pyrF* gene of *S. livingstonensis* Ac10 (*pyrF*^{Ac10}) in the pKKP plasmid[2] was replaced by the *pyrF*^{HM13} gene (*hm2280*) of *S. vesiculosa* HM13. A 699-bp DNA fragment containing the entire *pyrF*^{HM13} was amplified from the genomic DNA of *S. vesiculosa* HM13 by PCR with a primer set of *pyrF*^{HM13}-fw and *pyrF*^{HM13}-rv. Then, a 2.1-kbp fragment of pKKP, except for *pyrF*^{Ac10}, was amplified using a primer set of pKKP-fw and pKKP-rv. The two PCR products were purified and ligated using a NEBuilder HiFi DNA Assembly Cloning

System kit. The recombinant plasmid was introduced into *E. coli* S17-1/λ*pir*. Transformants were selected on LB plates containing 50 μg/mL Km. The construction of pKKPH was confirmed by diagnostic PCR using the primer set *pyrF*^{HM13}-fw and *pyrF*^{HM13}-rv.

***In vitro* binding assay between P49 and capsular polysaccharide (CPS)**

CPS was extracted from the cells of the P49-less mutant (Δp49Δ*pyrF*^{HM13}) by phenol-chloroform-petroleum ether method as previously reported[3]. Briefly, the cells grown at 18 °C were harvested at the stationary phase (OD_{600} = 2) and freeze-dried. Ten milligrams of the cells were mixed with one milli-litter of the phenol-chloroform-petroleum ether mixture (volume ratio of 2:5:8) and vortexed vigorously. After removing the cells by centrifugation at 10,000 ×*g* two times, the supernatant was subjected to nitrogen gas drying. By adding Milli Q water to the remaining phenol solution, lipopolysaccharide was precipitated and removed by centrifugation. The supernatant was dialyzed in one litter of Milli Q water overnight, and the concentrated CPS was collected. The concentration of the CPS extract was determined using a D-glucose solution and phenol-sulfuric acid method[4].

Purified P49 (450 μg) was incubated in the presence or absence of an equimolar amount of CPS (200 μg) in 1 mL of 0.1 M HEPES and 20 mM EDTA buffer (pH 7.4) at 4 °C for 1 h. The mixture was then subjected to gel filtration using a Superose 12 10/300 GL column (GE Healthcare, Little Chalfont, UK) at a flow rate of 0.4 mL/min with a fraction volume of 2.0 mL. Fractions were eluted with 0.1 M HEPES and 20 mM EDTA buffer (pH 7.4). A 10 μL aliquot of each fraction was analyzed by SDS-PAGE followed by staining with Alcian Blue and Coomassie Brilliant Blue (CBB) G-250 (Fig. 4). To evaluate the specificity of the P49–CPS interaction, a control experiment was performed using bovine serum albumin (BSA) (660 μg, corresponding to the same molar amount as 450 μg of P49) as a non-specific protein under the same conditions (Supplementary Fig. 4).

Supplementary Table 1. Predicted functions and localization of proteins encoded by the genes in the P49 gene cluster

Gene	Function	Localization**	Accession
hm3363	Wza, EpsE, polysaccharide export protein	PE	LC431043
hm3362	WecA, undecaprenyl-phosphate alpha-*N*-acetylglucosaminyl 1-phosphate transferase	ND	LC431042
hm3361	WecA, undecaprenyl-phosphate alpha-*N*-acetylglucosaminyl 1-phosphate transferase	IM	LC431041
hm3360	GspG2, Type II secretion system, major pseudopilin	IM	LC431040
hm3359	GspE2, Type II secretion system, secretion ATPase	CY	LC431039
hm3358	GspF2, Type II secretion system, inner membrane platform protein	IM	LC431038
hm3357	Pilin-like protein	ND	LC431037
hm3356	Pilin-like protein	ND	LC431036
hm3355	Pilin-like protein	ND	LC431035
hm3354	GspK2, Type II secretion system, minor pseudopilin	CY	LC431034
hm3353	PilN-like protein*, Type IV pilus assembly protein	ND	LC431033
hm3352	PilO-like protein*, Type IV pilus inner membrane component	ND	LC431032
hm3351	GspB2*, Type II secretion system, accessory protein	IM	LC431031
hm3350	Hypothetical protein, no significant similarity found	ND	LC431030
hm3349	GspD2, Type II secretion outer membrane pore forming protein, secretin	OM	LC431029
hm3348	Hypothetical protein, no significant similarity found	ND	LC431028
hm3347	P49, a major cargo protein of EMVs	EX	LC431027
hm3346	Hypothetical protein, no putative conserved domains	ND	LC431026
hm3345	GdpD, glycerophosphodiester phosphodiesterase	IM	LC431025
hm3344	Phosphoethanolamine transferase	IM	LC431024
hm3343	Wzx, a flippase to translocate extracellular polysaccharide precursors across inner membrane	IM	LC431023
hm3342	NfnB, nitroreductase family protein	CY	LC431022
hm3341	Ugd, UDP-glucose 6-dehydrogenase, provisional	CY	LC431021

* Estimated function based on structural prediction programs, I-TASSER (https://zhanggroup.org/I-TASSER/) and AlphaFold2 (https://colab.research.google.com/github/sokrypton/ColabFold/blob/main/AlphaFold2.ipynb)

** Protein localization was predicted with PSORTb version 3.0.3 (https://www.psort.org/psortb/).

EX, Extracellular space; OM, outer membrane; PE, Periplasm; IM, inner membrane; CY, cytoplasm; ND, not determined

Supplementary Table 2. Strains and plasmids used in this study

**Strains**	Description	Reference
Shewanella vesiculosa		
HM13-Rif^{r}	Rifampicin-resistant mutant of *S. vesiculosa* HM13, the parent strain of Δ*pyrF*^{HM13}.	5
Δ*pyrF*^{HM13}	Gene deletion mutant of *pyrF*^{HM13} (*hm2280*), a parent strain of gene deletion mutants	This study
Δ*p49*Δ*pyrF*^{HM13}	Deletion mutant of the gene coding for P49 (*hm3347,* LC431027) of Δ*pyrF*^{HM13}	This study
Δ*p49*Δ*pyrF*^{HM13}/p	Δ*p49*Δ*pyrF*^{HM13} harboring pJRD-Cm^{r}	This study
Δ*hm3343*Δ*p49*Δ*pyrF*^{HM13}	*hm3343* (*wzx,* LC431023)-deletion mutant of Δ*p49*Δ*pyrF*^{HM13}	This study
Δ*hm3343*Δ*p49*Δ*pyrF*^{HM13}/p	Δ*hm3343*Δ*p49*Δ*pyrF*^{HM13} harboring pJRD-Cm^{r}	This study
Δ*hm3343*Δ*p49*Δ*pyrF*^{HM13}/p*hm3343*	Δ*hm3343*Δ*p49*Δ*pyrF*^{HM13} harboring an expression vector of *wzx,* p*wzx*	This study
Δ*p49*	P49 gene::pKNOCK-Km^{r}	6
Δ*hm3359*	*hm3359* (*gspE2*)::pKNOCK-Km^{r}	6
Δ*hm3358*	*hm3358* (*gspF2*)::pKNOCK-Km^{r}	6
Δ*hm3354*	*hm3354* (*gspK2*)::pKNOCK-Km^{r}	6
Δ*hm3349*	*hm3349* (*gspD2*)::pKNOCK-Km^{r}	6
Δ*hm3345*	*hm3345* (*gdpD*)::pKNOCK-Km^{r}	6
Δ*hm3344*	*hm3344*::pKNOCK-Km^{r}	6
Δ*hm3343*	*hm3343* (*wzx*)::pKNOCK-Km^{r}	6
Δ*hm3342*	*hm3342* (*nfnB*)::pKNOCK-Km^{r}	6
Δ*hm3343/*p*hm3343*	Δ*hm3343* harboring an expression vector of *hm3343,* p*hm3343*	This study
Escherichia coli		
S17-1/λ*pir*	Plasmid donor cell for conjugative transformation of *S. vesiculosa* HM13 and its derivatives	7
**Plasmids**		
pKNOCK-Km^{r}	A gene knockout plasmid, RP4 *oriT* and R6K γ-*ori*; Km^{r}	8
pK*pyrF*^{HM13}	A gene knockout plasmid for *pyrF*^{HM13}	This study
pKKP	A gene knockout plasmid carrying *pyrF* of *S. livingstonensis* Ac10	2
pKKPH	A gene knockout plasmid carrying *pyrF* of *S. vesiculosa* HM13 (*pyrF*^{HM13})	This study
pKKPH-*p49*	*pyrF*^{HM13}-based gene-deletion plasmid of *p49*	This study
pKKPH-*hm3343*	*pyrF*^{HM13}-based gene-deletion plasmid of *hm3343*	This study
pJRD-Cm^{r}	A derivative of a broad host-range vector, pJRD215, carrying a chloramphenicol resistance gene	9
p*hm3343*	A gene complementation plasmid of *hm3343*, pJRD-Cm^{r} harboring *hm3343* and its predicted promoter region	This study

Supplementary Table 3. Primers used in this study

Primers	Description	Sequence (5´ to 3´)
Construction of pyrF-deletion mutant		
pyrF^{HM13}+1500-fw	Amplification of an upstream flanking region of *pyrF*^{HM13}	CTAGAACTAGTGGATCCCCCATGCTGGTCAGCCCAGTAAATTTCT
pyrF^{HM13}-del2		TGGAACTCCAGCGGTAATATGGTGTTAC
pyrF^{HM13}-del1	Amplification of a downstream flanking region of *pyrF*^{HM13}	ATATTACCGCTGGAGTTCCATTCTAGCAATGATGCCAGCAAAGTACAC
pyrF^{HM13}+1500-rv		ATATCGAATTCCTGCAGCCCTAGTCGGCATATGGATCATCAGCGC
pyrF^{HM13}-1.7up-fw	Confirmation of the genome insertion of pK*pyrF*^{HM13}	AGTATGTTCCACACTTTAAGCCGGG
pKNOCK-0.15dw-rv		CTACGTGTTCCGCTTCCTTTAGCA
Construction of pKKPH		
pyrF^{HM13}-fw	Amplification of *pyrF*^{HM13}	TAGCGTGATAACAAATCAAACATGTAGCGATTGGTAAATAGCCTTG
pyrF^{HM13}-rv		CCGCTGGAGTCCTAATGACTGAGAAATCAATTGTAGTCGCCC
pKKP-fw	Amplification of a linear fragment of pKKP excluding *pyrF*^{Ac10}	TTTGTTATCACGCTAACGGTTAGGTTGAC
pKKP-rv		TAGGACTCCAGCGGCAATAGCG
Construction of P49-gene-deletion mutant		
pKKPH-invfwd	Amplification of a linear fragment of pKKPH	GGGGGATCCACTAGTTCTAGAGCG
pKKPH-invrev		GGGCTGCAGGAATTCGATATCAAG
*p49*_upfwd	Amplification of an upstream flanking region of P49-coding gene	GAATTCCTGCAGCCCGGGTTAACGCTTGAAAAGCGAAAAAAAAG
*p49*_uprev		ACCATCTCCAATTTCATGAAATTTGGACTTACC
*p49*_downfwd	Amplification of a downstream flanking region of P49-coding gene	AAATTGGAGATGGTTCGTTTATAGAGGCTAATTTTTATCGGCTTATGGC
*p49*_downrev		ACTAGTGGATCCCCCAAGGAGAATAGTAAACAGGCGAATAACGC
Construction of hm3343-deletion mutant		
*wzx*_upfwd	Amplification of an upstream flanking region of *hm3343*	CTAGAACTAGTGGATCCCCCTGGGTCTATTGTATCCATAGAGGGCAAAC
*wzx*_uprev		TCAATTGTAAACCTTTGCCTAAAAAACACCT
*wzx*_downfwd	Amplification of a downstream flanking region of *hm3343*	AGGCAAAGGTTTACAATTGACCAATTTTACCTTAAAAAGGCGTGAGAAA
*wzx*_downrev		ATATCGAATTCCTGCAGCCCCTAAGATCGCCGACCACAACAATAGTACA
Confirmation of gene deletion		
Check_*pyrF*^{HM13}fwd	Confirmation of the deletion of *pyrF*^{HM13}	AGTATGTTCCACACTTTAAGCCGGG
Check_*pyrF*^{HM13}rev		GGCCAAGCAGGCTAATGCAG
Check_*p49*fwd	Confirmation of the deletion of the P49-coding gene	AGCAAGTGTAAGGATGTTTTTGCAGG
Check_*p49*rev		TAGCAACCACGCCGTAAATACACG
Check_*wzx*fwd	Confirmation of the deletion of *hm3343*	TAGGGTCTTGCACTACTGTCAGGTTTTTC
Check_*wzx*rev		CAAGCGCACCGTCTAAATAAATCAAATG
Complementation of hm3343-deletion mutant		
pJRD-invfwd	Amplification of a linear fragment of pJRD-Cm^{r}	CGTAATCCATGGATCAAGAG
pJRD-invrev		TAGTATAGTCTATAGTCCGTGG
*wzx*up500fwd	Amplification of *hm3343* and its upstream flanking region	CTCTTGATCCATGGATTACGCAGGGCGGTAATATGGTGAATTTTTTTATT
*wzx*rev		ACGGACTATAGACTATACTATCACTTGCCATTGAAGCACA
pJRD_CheckFW1	Confirmation of insertion of *hm3343* into pJRD-Cm^{r}	GTGCGCCAACTACCAGCT
*wzx*_CheckFw2		GTGATATTATCTGCGGTATCGAAATTTATGC
pJRD_CheckRV		GCCTGACTGCGTTAGCAATTTAAC

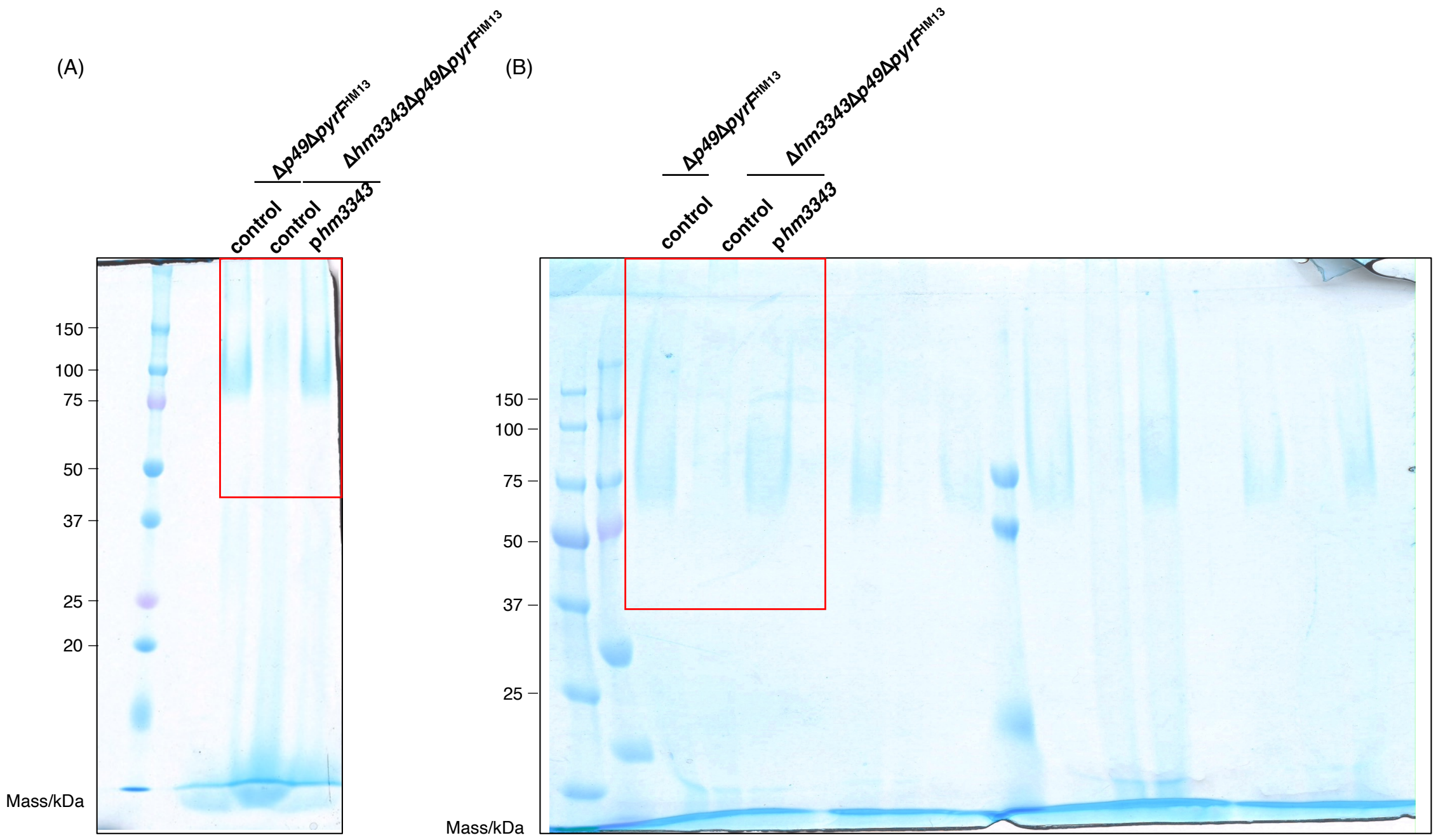

Supplementary Fig. 1 Full-length gels corresponding to Fig. 2A and B.
The cropped areas used in the main figure are indicated by red boxes.

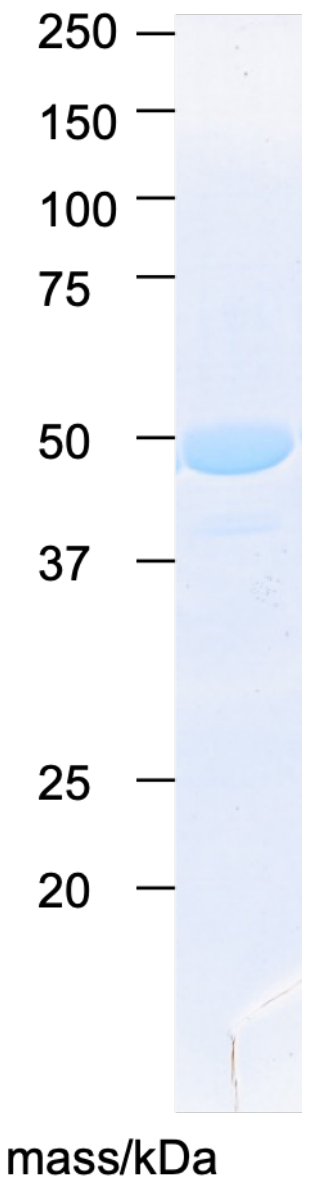

Supplementary Fig. 2 Purity of P49 used for the *in vitro* assay
SDS-PAGE analysis of purified P49 (2 μg) stained with CBB G-250.

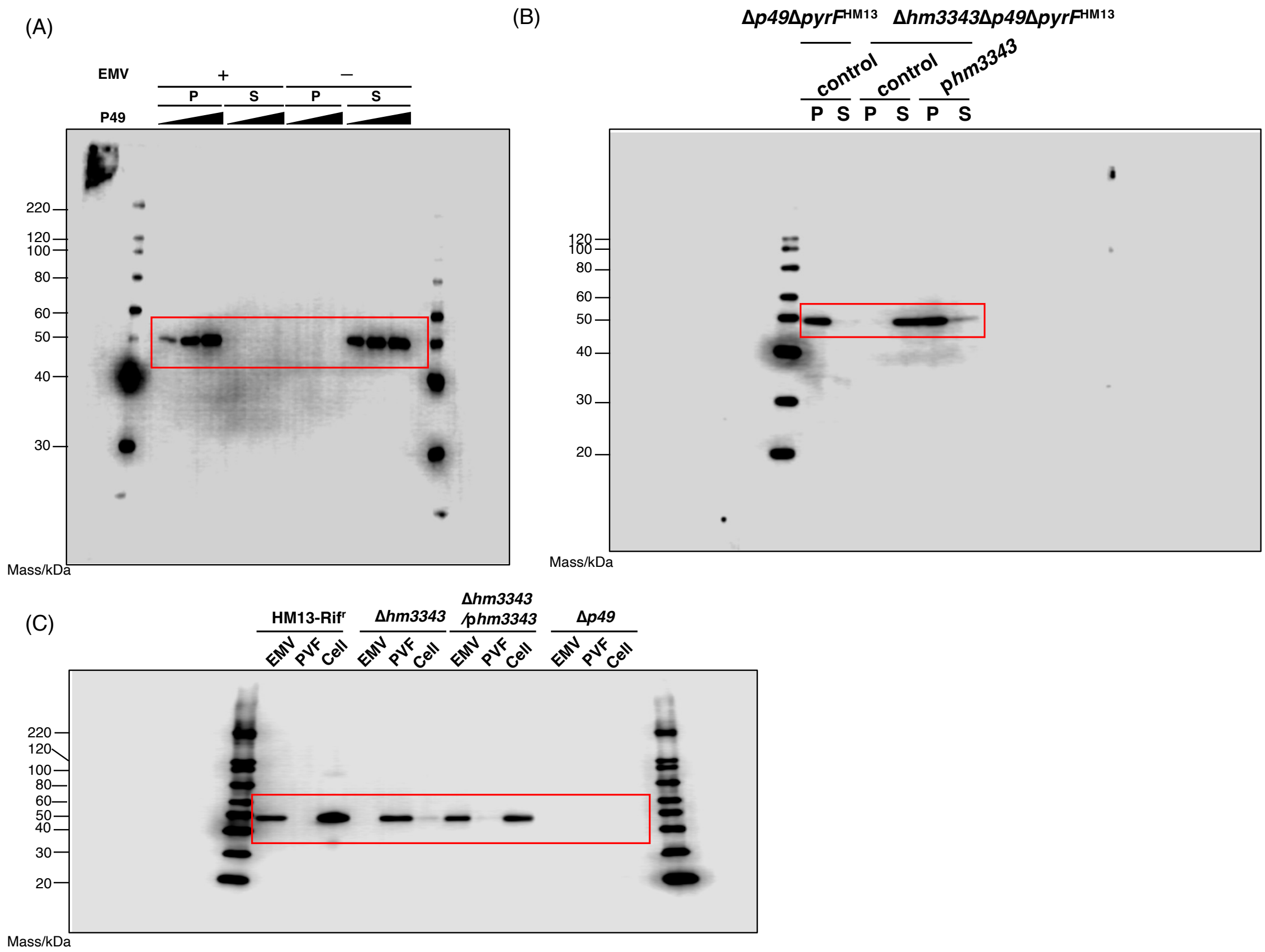

Supplementary Fig. 3 Full-length blots corresponding to Fig. 3A-C.
The cropped areas used in the main figures are indicated by red boxes.

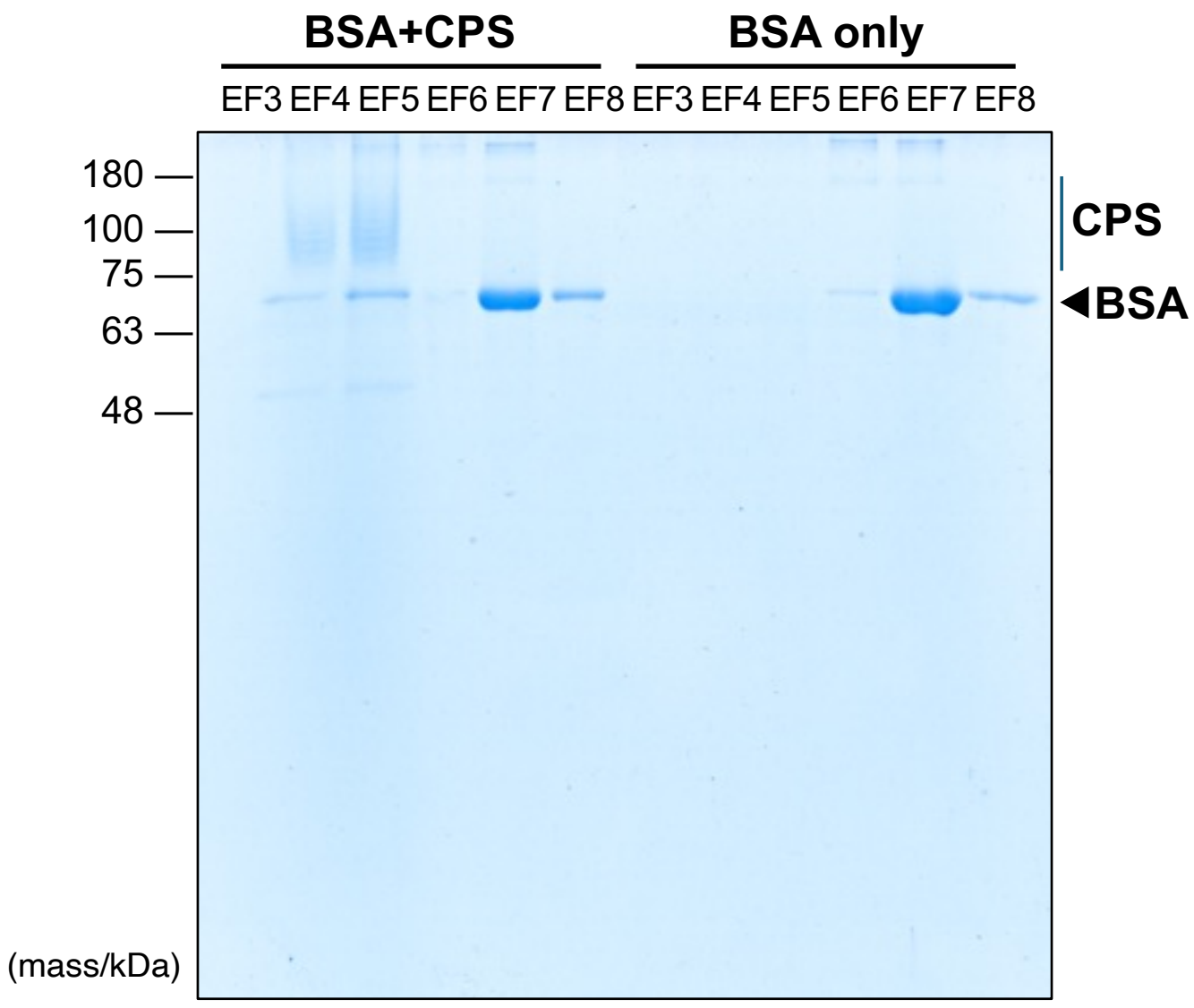

Supplementary Fig. 4 *In vitro* binding capacity of bovine serum albumin to CPS
SDS-PAGE analysis of elution fractions from gel-filtration chromatography. Bovine serum albumin (BSA) was incubated in the presence (BSA + CPS) or absence (BSA only) of CPS extracted from the cells of the Δp49Δ*pyrF* HM13 mutant. The mixtures were fractionated using a Superose 12 10/300 GL column, and the elution fractions 3–8 (EF3–EF8) were subjected to SDS-PAGE followed by CBB G-250 staining. The positions of CPS and BSA are shown in the figure.

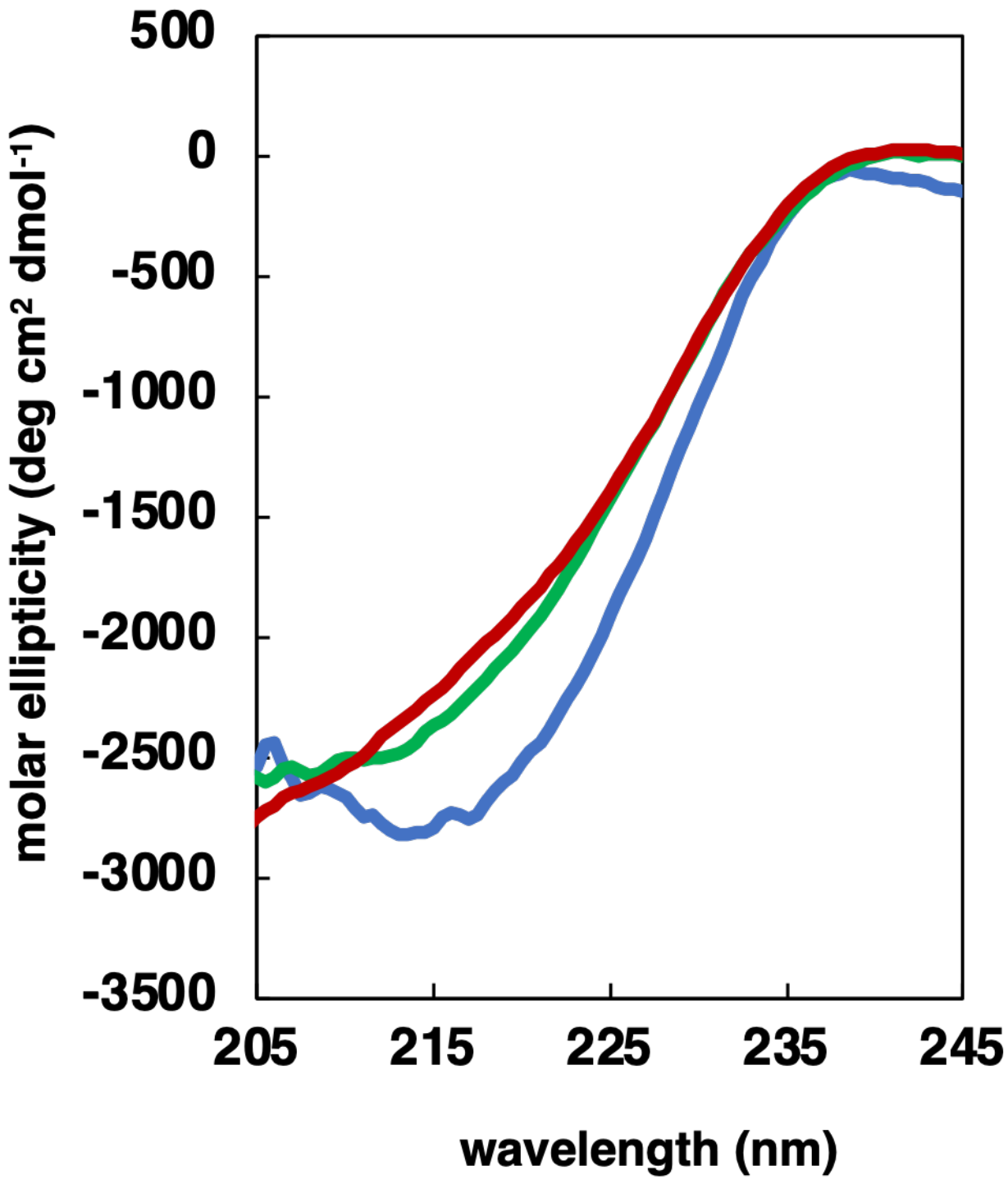

Supplementary Fig. 5 The CD spectra of the EMV-free purified P49 (red) and P49 incubated with the EMVs from Δ*p49* (blue) and Δ*hm3343* (green). The background CD signals from EMVs alone were subtracted from the spectra of samples containing EMVs.

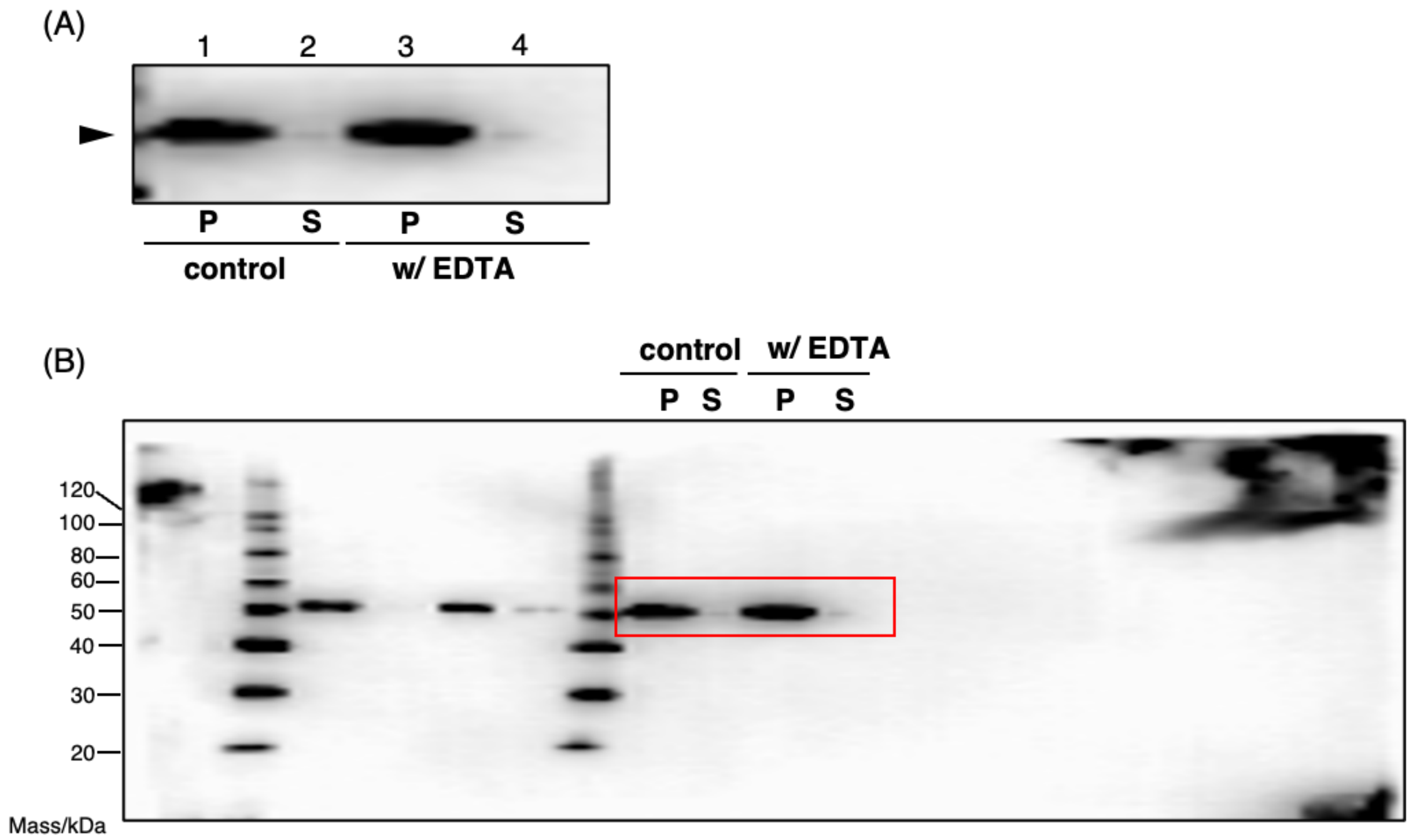

Supplementary Fig. 6 Effects of EDTA supplementation on *in vitro* P49 binding to EMVs.

(A) *In vitro* P49-binding assays were performed in the presence (w/ EDTA) or absence (control) of 20 mM EDTA. Samples were then fractionated into pellet (P) and supernatant (S) by ultracentrifugation and analyzed by western blotting with an anti-P49 antibody. Cropped from the original full-length blots shown in (B). The cropped area is indicated by the red box.

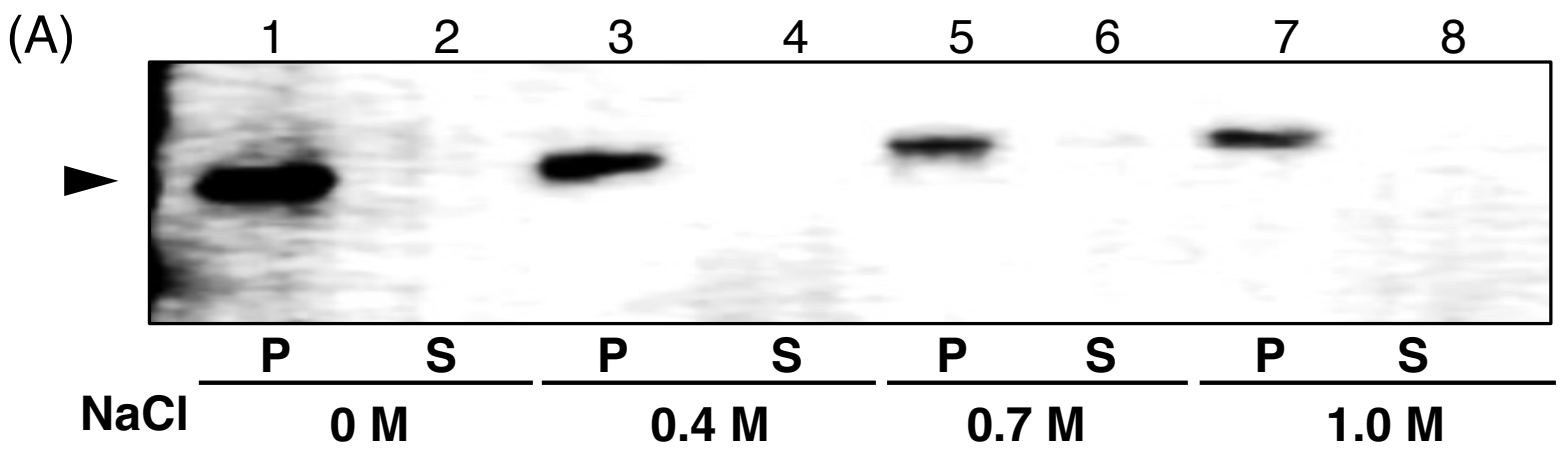

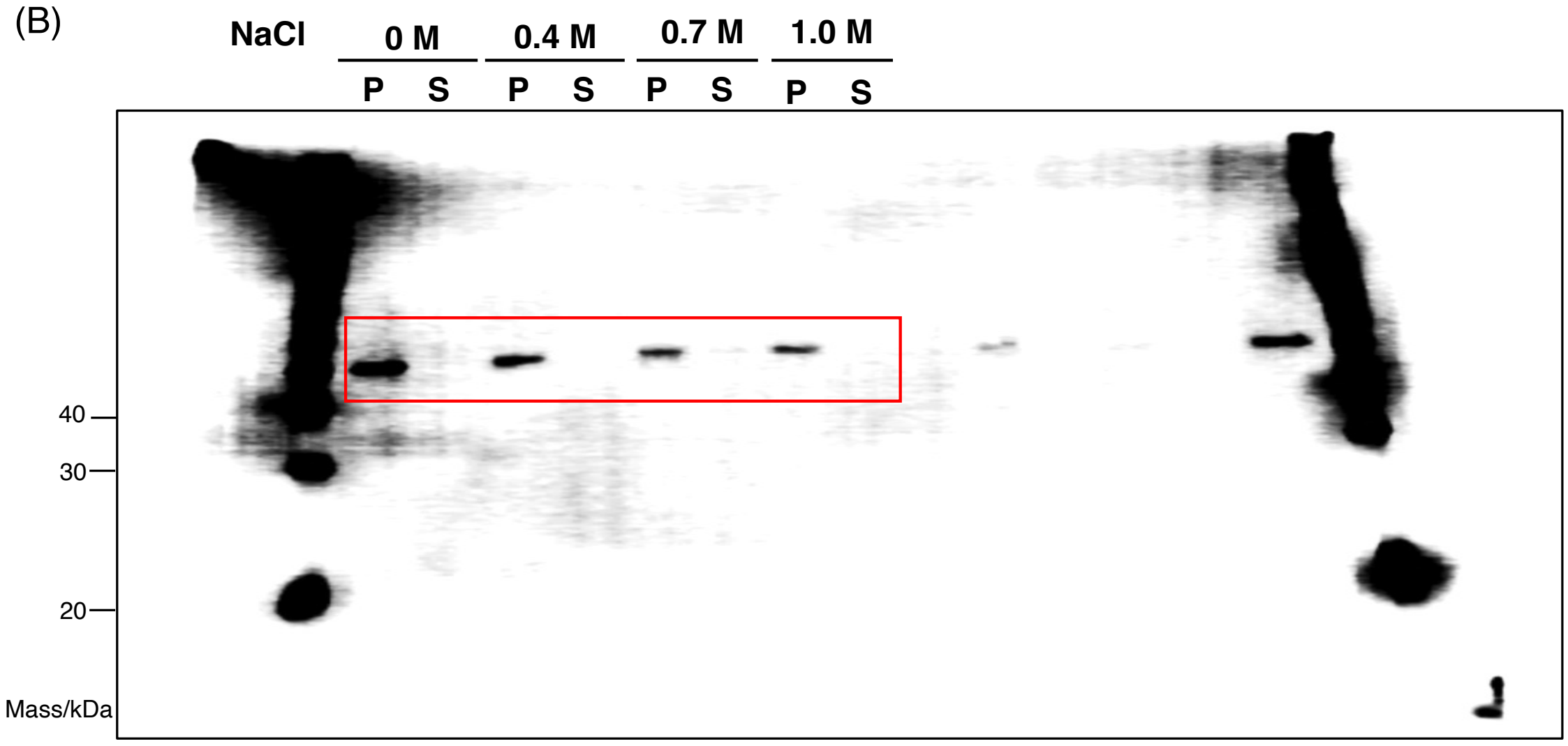

Supplementary Fig. 7 Effects of ionic strength on *in vitro* P49 binding to EMVs. (A) *In vitro* P49-binding assays were performed at various NaCl concentrations (0, 0.4, 0.7, and 1.0 M). Following incubation, samples were fractionated into pellet (P) and supernatant (S) by ultracentrifugation and analyzed by western blotting. The results were confirmed in three independent experiments. Cropped from the original full-length blots shown in (B). The cropped area is indicated by the red box.

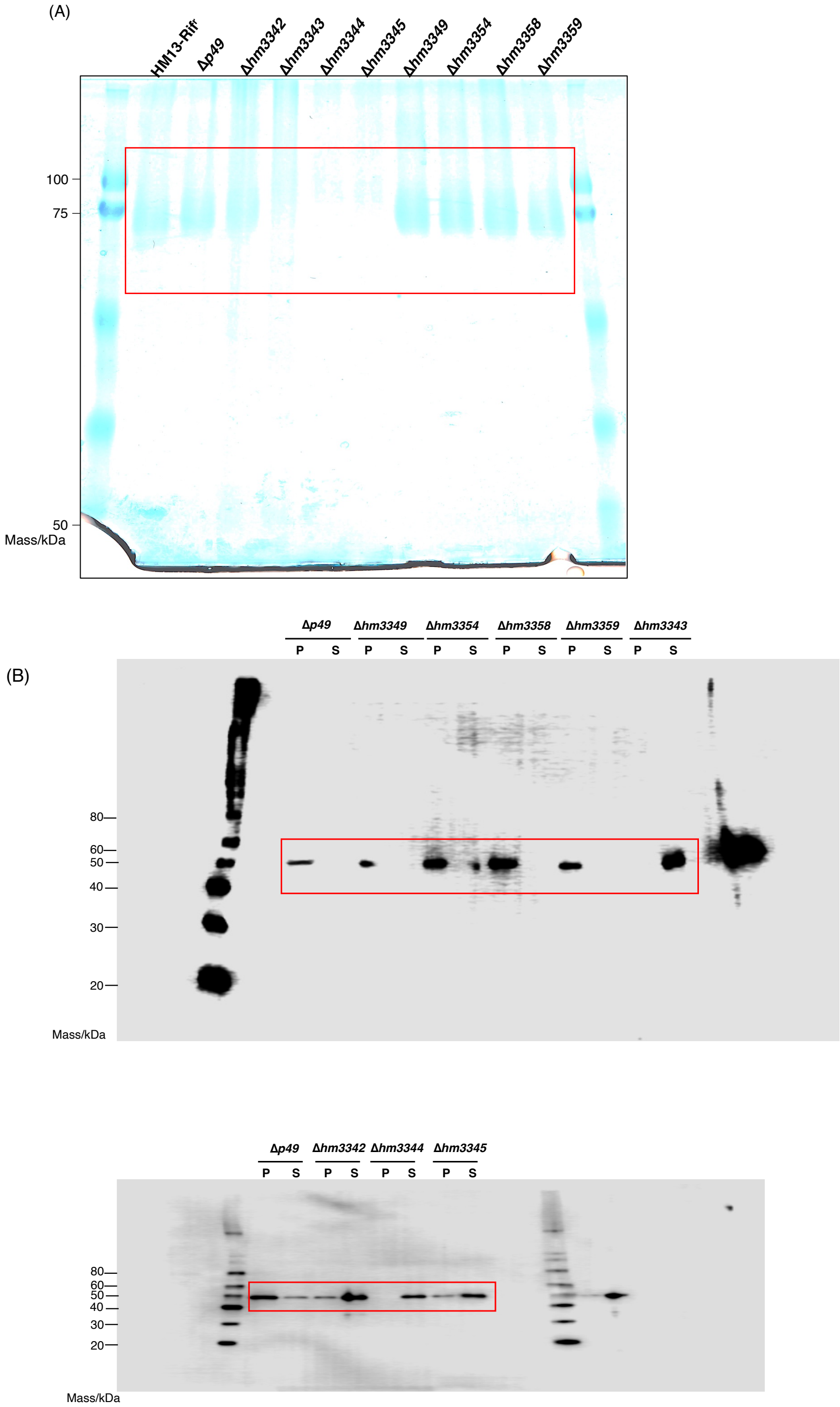

Supplementary Fig. 8 Full-length gel and blots corresponding to Fig. 6A and B. The cropped areas used in the main figures are indicated by red boxes.

References

1. Kawamoto, J. & Kurihara, T. Membrane vesicles produced by *Shewanella vesiculosa* HM13 as a prospective platform for secretory production of heterologous proteins at low temperatures. *Methods Mol. Biol.* **2414**, 191–205 (2022).
2. Ito, T., Gong, C., Kawamoto, J. & Kurihara, T. Development of a versatile method for targeted gene deletion and insertion by using the *pyrF* gene in the psychrotrophic bacterium, *Shewanella livingstonensis* Ac10. *J. Biosci. Bioeng.* **122**, 645–651 (2016).
3. Galanos, C., Lüderitz, O. & Westphal, O. A new method for the extraction of R lipopolysaccharides. *Eur. J. Biochem.* **9**, 245–249 (1969).
4. Nielsen, S. S. Phenol-sulfuric acid method for total carbohydrates. in *Food Analysis Laboratory Manual* (ed. Nielsen, S. S.) 47–53 (Springer US, Boston, MA, 2010).
5. Chen, C. *et al.* Isolation of a novel bacterial strain capable of producing abundant extracellular membrane vesicles carrying a single major cargo protein and analysis of its transport mechanism. *Front. Microbiol.* **10**, 3001 (2019).
6. Kamasaka, K. *et al.* Genetic characterization and functional implications of the gene cluster for selective protein transport to extracellular membrane vesicles of *Shewanella vesiculosa* HM13. *Biochem. Biophys. Res. Commun.* **526**, 525–531 (2020).
7. Simon, R., Priefer, U. & Pühler, A. A broad host range mobilization system for *in vivo* genetic engineering. Transposon mutagenesis in Gram negative bacteria. *Biotechnology* **1**, 784–791 (1983).
8. Alexeyev, M. F. The pKNOCK series of broad-host-range mobilizable suicide vectors for gene knockout and targeted DNA insertion into the chromosome of Gram-negative bacteria. *Biotechniques* **26**, 824–828 (1999).
9. Toyotake, Y., Cho, H.-N., Kawamoto, J. & Kurihara, T. A novel 1-acyl-*sn*-glycerol-3-phosphate *O*-acyltransferase homolog for the synthesis of membrane phospholipids with a branched-chain fatty acyl group in *Shewanella livingstonensis* Ac10. *Biochem. Biophys. Res. Commun.* **500**, 704–709 (2018).
